# Liquid Nitrogen Sources Assisting Gram‐Scale Production of Single‐Atom Catalysts for Electrochemical Carbon Dioxide Reduction

**DOI:** 10.1002/advs.202205639

**Published:** 2023-02-15

**Authors:** Beibei An, Jingsheng Zhou, Liangjing Duan, Xiao Liu, Guanyao Yu, Tiegang Ren, Xugeng Guo, Yuanyuan Li, Hans Ågren, Li Wang, Jinglai Zhang

**Affiliations:** ^1^ Henan Province Engineering Research Center of Green Anticorrosion Technology for Magnesium Alloys Henan University Kaifeng Henan 475004 P. R. China; ^2^ Henan Engineering Research Center of Corrosion and Protection for Magnesium Alloys Henan University Kaifeng Henan 475004 P. R. China; ^3^ College of Chemistry and Chemical Engineering Henan University Kaifeng Henan 475004 P. R. China

**Keywords:** CO_2_ reduction, gram‐scale strategy, ionic liquids, Ni single atom, nitrogen sources

## Abstract

Developing metal‐nitrogen‐carbon (M‐N‐C)‐based single‐atom electrocatalysts for carbon dioxide reduction reaction (CO_2_RR) have captured widespread interest because of their outstanding activity and selectivity. Yet, the loss of nitrogen sources during the synthetic process hinders their further development. Herein, an effective strategy using 1‐butyl‐3‐methylimidazolium tetrafluoroborate ([BMIM][BF_4_]) as a liquid nitrogen source to construct a nickel single‐atom electrocatalyst (Ni‐SA) with well‐defined Ni‐N_4_ sites on a carbon support (denoted as Ni‐SA‐BB/C) is reported. This is shown to deliver a carbon monoxide faradaic efficiency of >95% over a potential of −0.7 to −1.1 V (vs reversible hydrogen electrode) with excellent durability. Furthermore, the obtained Ni‐SA‐BB/C catalyst possesses higher nitrogen content than the Ni‐SA catalyst prepared by conventional nitrogen sources. Importantly, only thimbleful Ni nanoparticles (Ni‐NP) are contained in the large‐scale‐prepared Ni‐SA‐BB/C catalyst without acid leaching, and with only a slight decrease in the catalytic activity. Density functional theory calculations indicate a salient difference between Ni‐SA and Ni‐NP in the catalytic performance toward CO_2_RR. This work introduces a simple and amenable manufacturing strategy to large‐scale fabrication of nickel single‐atom electrocatalysts for CO_2_‐to‐CO conversion.

## Graphical abstract

The Ni‐N‐C‐based single‐atom electrocatalyst (Ni‐SA‐BB/C) is synthesized with [BMIM][BF_4_] as a liquid nitrogen source via one‐step pyrolytic strategy for carbon dioxide reduction reaction, achieving excellent selectivity with high FE_CO_ values of >95% throughout a wide potential of −0.8 to −1.1 V (vs reversible hydrogen electrode) and durability.

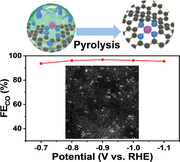

## 1 Introduction

The excessive consumption of fossil fuels has led to dramatic climate change and serious energy crises.^[^ ^1^ ^]^ Electroreduction of carbon dioxide (CO_2_) is a favorable strategy to obtain value‐added products and to achieve carbon‐neutral energy circulation via renewable energy sources.^[^ ^2^ ^]^ However, CO_2_ reduction reaction (CO_2_RR) still suffers from the competitive reduction of water itself to hydrogen and the intrinsic sluggish kinetics of CO_2_ molecules, which result in low conversion efficiency and selectivity.^[^ ^3^, ^4^, ^5^ ^]^ It is highly desirable to develop an electrocatalyst that can achieve high activity and selectivity. The single‐atom (SA) electrocatalysts display the exceptional catalytic performance due to the maximum atom utilization, well‐exposed active sites, and unique electronic properties.^[^ ^6^, ^7^, ^8^, ^9^, ^10^ ^]^ The SA electrocatalysts with the metal‐nitrogen‐carbon (M‐N‐C) unit have attracted considerable attentions since the unique coordination environment endows M‐N‐C with excellent activity. Since the pioneering report of the M‐N‐C based SA electrocatalyst in 2015,^[^ ^11^ ^]^ most efforts have focused on constructing more efficient SA electrocatalysts for CO_2_RR with different metal center including Fe, Co, Mn, Zn, and Sn.^[^ ^12^, ^13^, ^14^, ^15^ ^]^ However, the appearance of Ni‐N‐doped carbon materials experiences some difficulties because the Ni—N bonds are more susceptible to rupture under high‐temperature treatment. After the successful synthesis of Ni—N‐modified graphene by Kamiya et al.,^[^ ^16^ ^]^ Ni‐N‐C‐based SA electrocatalysts get into vigorous advancement.^[^ ^17^, ^18^ ^]^ As compared with the variation of the metal precursors, less attention has been directed on the exploration of the N precursors.

Actually, N species play a vitally important role in the formation of M‐N‐C moieties to improve the intrinsic activity of the catalysts. On the one hand, the introduction of electron‐rich N into the carbon network can break the intrinsic delocalized *π*‐system of the carbon material leading to an enhanced electron density in the surrounding region of metal center, which is a critical aspect to prompt the CO_2_RR.^[^ ^19^ ^]^ Meanwhile, doping with N also creates extra active sites on the carbon support.^[^ ^20^ ^]^ On the other hand, the introduced N serves as a bridge that links the metal atom and carbon atom in the support to form the M‐N‐C moieties. To date, it is still challenging to maximize the generation of M‐N‐C moieties in SA electrocatalysts due to the inevitable loss of N sources during the high‐temperature synthetic process.^[^ ^21^ ^]^

Gas N and solid N sources are two popular sources used to fabricate M‐N‐C‐based SA electrocatalysts.^[^ ^22^, ^23^ ^]^ For gas N sources, normally NH_3_, the loss of nitrogen is almost inevitable due to the continuous injection and discharge of NH_3_ during the process of synthesis.^[^ ^24^ ^]^ For solid N sources (normally melamine, dicyandiamide, dimethylimidazole, or urea), it is difficult to form homogenously mixed precursors. This is a nonnegligible factor leading to agglomeration in the final synthesized sample. Introduction of a liquid environment is an effective strategy to avoid the above issues. Unfortunately, common liquid environment makers, like molten salt (KCl or NaCl), can only become liquid over 800 °C.^[^ ^25^, ^26^ ^]^ A liquid N source at room temperature would make it very much possible to retard the loss of N sources and minimize the agglomeration. Here, ionic liquids (ILs) are potential N sources being in liquid phase at room temperature.^[^ ^27^ ^]^ More importantly, the space confinement effect supplied by ILs is favorable to form the SA site.^[^ ^28^ ^]^ In addition, ILs can also offer a strong polarity to impede the formation of metal—metal bonds, which is highly desirable for the formation of atomically dispersed metal sites and for eventually achieving large‐scale preparation of the catalysts.^[^ ^29^ ^]^ Benefiting from these merits, ILs are anticipated to be promising N sources to prepare M‐N‐C‐based SA catalysts. In recent years, ILs have been applied as a reaction medium to prepare SA catalysts or as electrolyte additives in some electrocatalytic reactions.^[^ ^30^, ^31^, ^32^ ^]^ However, the unique roles of ILs as liquid N sources to synthesize SA catalysts have not been much exploited so far.

In this work, 1‐butyl‐3‐methylimidazolium tetrafluoroborate ([BMIM][BF_4_]) was employed as N precursor to prepare nickel single‐atom catalysts distributed in a carbon support (Ni‐SA‐BB/C) via a simple one‐step pyrolysis for the electrocatalytic reduction of CO_2_ to carbon monoxide (CO). For comparison, Ni‐SA dispersed on the carbon support with the traditional N precursors, dicyandiamide (denoted as Ni‐SA‐DCD/C), or melamine (denoted as Ni‐SA‐MA/C), were also prepared following the same fabrication process. The obtained Ni‐SA‐BB/C possesses higher N content (5.0 wt%) relative to Ni‐SA‐DCD/C (2.4 wt%) and Ni‐SA‐MA/C (2.0 wt%) suggesting an enhanced amount of loaded Ni‐SA in the Ni‐SA‐BB/C. When Ni‐SA‐BB/C serves as a catalyst, it is here shown to provide an excellent CO_2_ electro‐reduction performance with nearly 100% selectivity toward CO within a wide potential range. In addition, the great applicability of this strategy was further validated by altering the ILs (e.g., 1‐butyl‐3‐methylimidazolium hexafluorophosphate [BMIM][PF_6_] and 1‐ethyl‐3‐methylimidazolium tetrafluoroborate [EMIM][BF_4_]) and loading different metal single atoms (e.g., Co, Cu, Fe, and Sn). Importantly, the large‐scale prepared Ni‐SA‐BB/C (denoted as Ni‐SA‐BB/C‐G) without acid leaching still maintains excellent catalytic activity. The difference in catalytic activity between Ni‐SA‐BB/C and Ni‐SA‐BB/C‐G was also elucidated by first principle calculations. This work supplies a gram‐scale strategy to construct Ni‐SA catalysts and reveals the vital importance of ILs as liquid N precursors toward improved catalysis.

## 2 Results and Discussion

### 2.1 Synthesis and Characterization of Ni‐SA‐BB/C

**Scheme 1:**
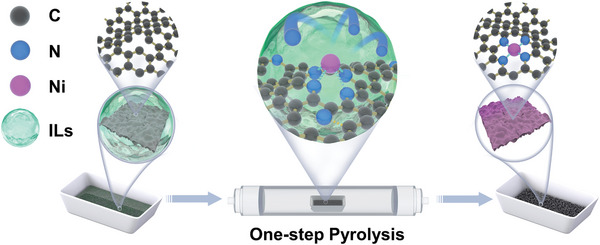
Schematic illustration of the preparation route for Ni‐SA‐BB/C.

**Figure 1:**
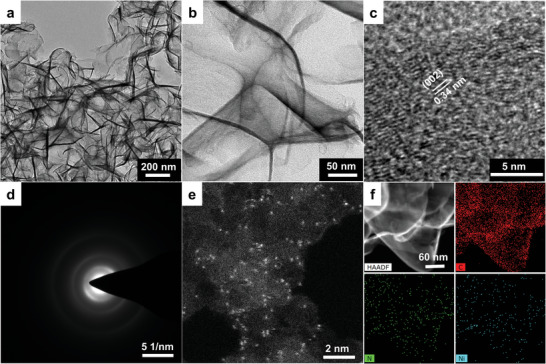
Electron microscopy characterizations of as‐synthesized Ni‐SA‐BB/C. a) Low‐magnification TEM image. b) High‐magnification TEM image. c) HR‐TEM image. d) SAED pattern. e) Aberration‐corrected HAADF‐STEM image. f) Dark‐field TEM image and EDS elemental mapping images.

The simple synthetic strategy was illustrated in **Scheme** **1**. First, the sodium citrate was carbonized to obtain carbon support. Then, the carbon support was dispersed in [BMIM][BF_4_] (N precursor) containing NiCl_2_ (Ni precursor). Finally, the mixture was pyrolyzed under the protection of Ar flow, during which the Ni and N were embedded into the carbon support to form a Ni‐N‐C structure. Scanning electron microscope and transmission electron microscope (TEM) images of the carbon support show a 2D nanostructure (Figure S1, Supporting Information). The carbon support was pyrolyzed in [BMIM][BF_4_] containing NiCl_2_, without affecting its 2D structure. TEM images at different magnifications reveal that the obtained graphene‐like Ni‐SA‐BB/C show rich wrinkles and ripples but no aggregated Ni nanoparticles (Ni‐NP) (**Figure** **1**a,b). Such morphology features may be beneficial for CO_2_ molecular accessibility.^[^ ^33^ ^]^ In addition, only short‐range ordered graphitic domains with a lattice spacing of 0.34 nm can be observed in the high‐resolution TEM (HR‐TEM) image (Figure 1c), which is in good agreement with a (002) plane of graphene, suggesting that the degree of graphitization of the carbon support is low. The selected area electron diffraction (SAED) pattern confirms its poor crystallinity (Figure 1d). The aberration‐corrected high angle annular dark‐field scanning transmission electron microscopy (HAADF‐STEM) image of Ni‐SA‐BB/C in Figure 1e exhibits that the isolated bright spots are Ni atoms implying the possible existence of isolated Ni‐SA catalysts. The energy‐dispersive X‐ray spectroscopy (EDS) mapping images further reveal that Ni and N are homogenously dispersed in the carbon support (Figure 1f). The Ni content in Ni‐SA‐BB/C is about 0.53 wt% according to the inductively coupled plasma mass spectroscopy (ICP‐MS).

The X‐ray diffraction (XRD) patterns of Ni‐SA‐BB/C display a broad peak at around 24° ascribing to the (002) plane of the graphic carbon (Figure S2a, Supporting Information),^[^ ^34^, ^35^ ^]^ which is consistent with the HR‐TEM result. No diffraction peaks of Ni‐NP and its oxides are observed in the XRD pattern of the Ni‐SA‐BB/C, further demonstrating that there is no impurity in the Ni‐SA‐BB/C sample. Raman spectra of the carbon support as well as of Ni‐SA‐BB/C exhibit two peaks, at 1349 and 1598 cm^−1^ (Figure S2b, Supporting Information), respectively, assigned to disordered sp^3^ carbon (D‐band) and graphite sp^2^ carbon (G‐band). The relative intensity ratio of D to G‐band (*I*_D_ */I*_G_) slightly increases from 0.91 for the carbon support to 0.96 for Ni‐SA‐BB/C, which may be due to the introduced Ni and N leading to an increased degree of disorder.^[^ ^36^ ^]^ In addition, N_2_ adsorption–desorption measurements verify that Ni‐SA‐BB/C possesses a large surface area (124.1 m^2^ g^−1^) with a highly porous feature (Figure S3, Supporting Information). This feature is beneficial for the exposure of catalytic sites and the substrates/product transfer.^[^ ^37^ ^]^

### 2.2 Atomic Structure Analysis of Ni‐SA‐BB/C

**Figure 2:**
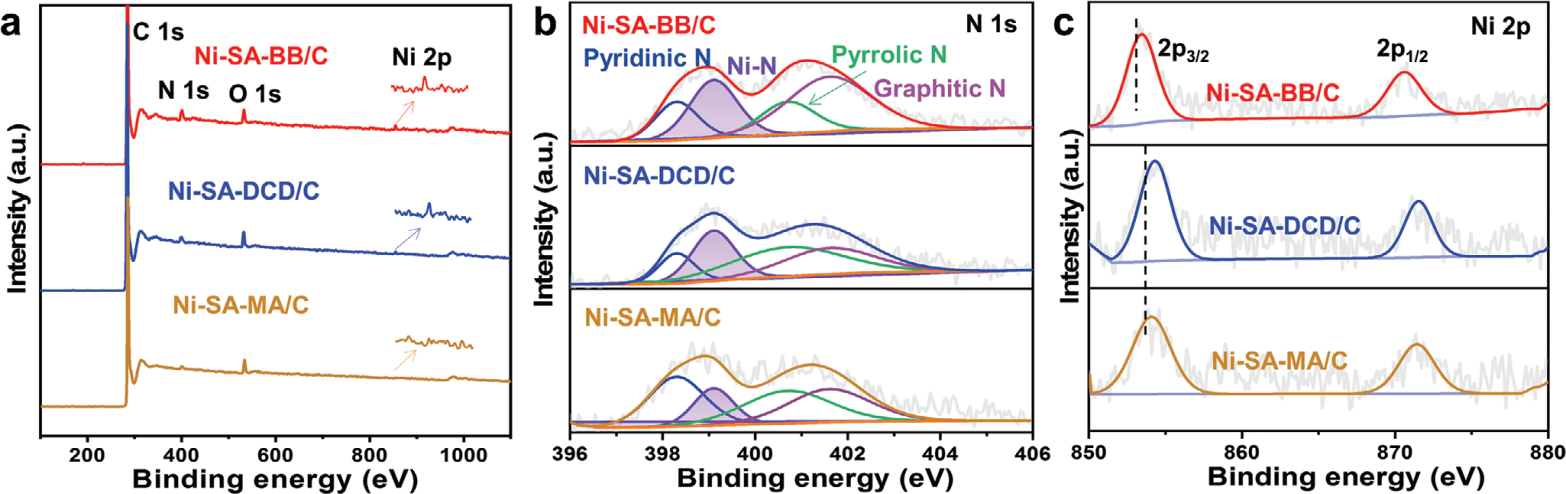
Structure characterizations of Ni‐SA‐BB/C. a) Full XPS spectra, b) N 1s HR‐XPS spectra, c) Ni 2p HR‐XPS spectra of Ni‐SA‐BB/C, Ni‐SA‐DCD/C, and Ni‐SA‐MA/C, respectively.

Subsequently, the local atomic environment of Ni‐SA in the Ni‐SA‐BB/C was explored via X‐ray photoelectron spectroscopy (XPS) and synchrotron‐radiation‐based X‐ray absorption spectroscopy (XAS). To obtain the surface compositions and chemical states of the elements presenting in Ni‐SA‐BB/C, XPS was analyzed. For comparison, Ni‐SA‐DCD/C and Ni‐SA‐MA/C were prepared separately using DCD and MA as the N precursors (the detailed experimental process is given in the Supporting Information). Full‐scan XPS spectra of three samples (Ni‐SA‐BB/C, Ni‐SA‐DCD/C, and Ni‐SA‐MA/C) demonstrate the existence of the relevant elements (**Figure** **2**a). In the high‐resolution XPS (HR‐XPS) N 1s spectra for three samples, an asymmetric band appears that can be deconvoluted into four peaks (Figure 2b). Three peaks located at 398.3, 400.7, and 401.4 eV are assigned to pyridinic N, pyrrolic N, and graphitic N, respectively.^[^ ^38^ ^]^ Another peak at 399.1 eV corresponds to a distinct Ni—N bond.^[^ ^39^ ^]^ It is notable that the content of Ni—N in Ni‐SA‐BB/C (25.8%) is larger relative to that in Ni‐SA‐DCD/C (18.7%) and Ni‐SA‐MA/C (15.1%), implying the high content of N in Ni‐SA‐BB/C sample. Based on XPS results, indeed, Ni‐SA‐BB/C has a significantly higher content of N (5.03 wt%) in comparison with the other two samples (Ni‐SA‐DCD/C 2.4 wt% and Ni‐SA‐MA/C 2.03 wt%). These results imply that the employment of [BMIM][BF_4_] as a liquid N source can effectively prevent the loss of N and promote the load of Ni species. We refer here to the high‐resolution Ni 2p electron core‐level XPS spectra of Ni‐SA‐BB/C, Ni‐SA‐DCD/C, and Ni‐SA‐MA/C shown in Figure 2c. Two main peaks observed from Ni‐SA‐BB/C at the binding energy positions of 853.4 and 870.6 eV correspond to the Ni 2p_3/2_ and Ni 2p_1/2_ levels, respectively, indicating a shift to the lower binding energy with respect to that of Ni‐SA‐DCD/C (854.3 and 871.5 eV) and Ni‐SA‐MA/C (854.1 and 871.4 eV). The rich content of N species in Ni‐SA‐BB/C can be expected to induce a variation of electron density distribution toward the Ni active sites ultimately leading to the observed shift of the binding energy peak.^[^ ^40^, ^41^ ^]^ The electron enrichment of the Ni active sites can improve their capability to donate electrons to the reactants, enhancing the electrochemical performance of the reduction reaction.^[^ ^42^ ^]^ In addition, the peaks of Ni 2p_3/2_ for three samples locate between reported Ni^0^ (853.0 eV) and Ni^2+^ (855.7 eV)^[^ ^43^ ^]^ indicating the presence of Ni^*δ*+^ (0 < *δ* < 2) centers in them.

**Figure 3:**
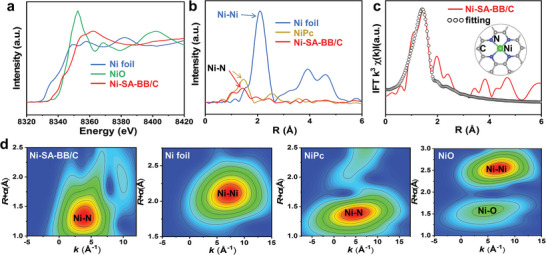
a) Ni *K*‐edge XANES spectra of as‐prepared samples and references. b) Fourier transformed curves of Ni *K*‐edge EXAFS spectra. c) Ni *K*‐edge EXAFS fitting result of Ni‐SA‐BB/C, inset shows the proposed coordination configuration of the Ni center. d) The wavelet transform of as‐prepared samples and references.

To further shed some light on the chemical environment of the Ni atoms, more detailed atomic structure information was obtained from synchrotron‐radiation‐based XAS, which consists of X‐ray absorption near‐edge structure (XANES) and extended X‐ray absorption fine structure (EXAFS). **Figure** **3**a displays the Ni *K*‐edge XANES spectra of Ni‐SA‐BB/C with Ni foil and NiO as references. The rising edge of Ni‐SA‐BB/C is positioned between that of the Ni foil and NiO, indicating that the valence of the Ni atom is between 0 and +2.^[^ ^23^ ^]^ This observation also agrees well with the fitting results of Ni 2p XPS spectra. The Fourier‐transformed *K_3_*‐weighted EXAFS in *R* space confirms that the dominant characteristic peak for the Ni‐SA‐BB/C locates at 1.4 Å, which is in accordance with the peak in the standard nickel phthalocyanine (NiPc) reference (1.4 Å), suggesting the formation of a Ni—N bond sample,^[^ ^10^ ^]^ as shown in Figure 3b. Meanwhile, the absence of Ni—Ni bond at 2.1 Å demonstrates the nonexistence of Ni‐NP. Based on the fitting results of the EXAFS curves, the coordination number of Ni—N in Ni‐SA‐BB/C is 3.9 (Figure 3c and Table S1, Supporting Information). Hence, we can conclude that the local geometric structure of Ni‐SA‐BB/C is accurately confirmed to be the chemical configuration of a single Ni atom coordinated with four N atoms. A wavelet transform (WT) providing both the *K* and *R* space resolution was employed to further confirm the above conclusion. In Figure 3d, the WT contour plot of Ni‐SA‐BB/C shows a strong signal with an intensity maximum at ≈ 3.4 Å^−1^, similar to that in NiPc. Furthermore, no Ni—Ni and Ni—O signals are observed. On the whole, these results strongly prove that the atomic dispersion of Ni was anchored by four‐coordinating N atoms in the carbon support. To certify the generality of the strategy, other metal‐SA (Co‐SA, Cu‐SA, Fe‐SA, and Sn‐SA) samples were prepared with the same precursor and carbon support. The characterization results imply that this sample strategy can be easily extended to other metal‐SAs (Figure S4 and Table S2, Supporting Information).

### 2.3 Electrocatalytic Performance for CO_2_RR

**Figure 4:**
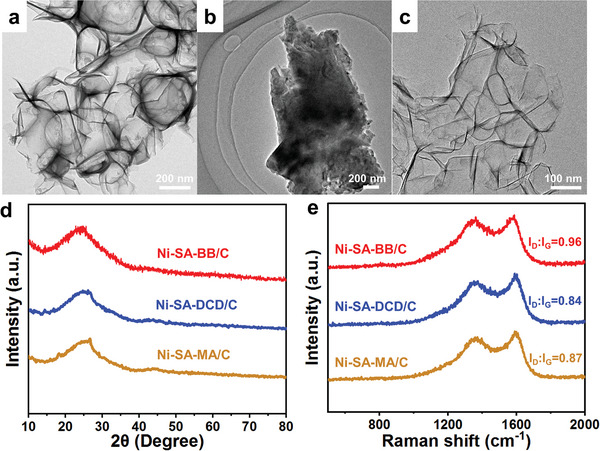
TEM images of a) Ni‐SA‐BB/C, b) Ni‐SA‐DCD/C, and c) Ni‐SA‐MA/C. d) XRD patterns, e) Raman patterns of Ni‐SA‐BB/C, Ni‐SA‐DCD/C, and Ni‐SA‐MA/C.

Electrochemical reduction of CO_2_ on the fabricated catalysts described above was measured in a gas‐tight H‐type electrochemical cell separated with a Nafion‐117 membrane. Initially, the effect of pyrolysis temperature on the property and electrochemical CO_2_ reduction performance of the samples was investigated with temperature varying from 900 to 1100 °C. The synthesized samples are denoted as Ni‐SA‐BB/C (900), Ni‐SA‐BB/C (1000), and Ni‐SA‐BB/C (1100), respectively. The results of Raman (Figure S5a, Supporting Information), XPS (Figure S5b–d, Supporting Information), and electrocatalytic performance (Figure S6, Supporting Information), indicate that 1000 °C is the optimal pyrolysis temperature for the as‐prepared Ni‐SA‐BB/C catalyst toward electrochemical CO_2_ reduction. Subsequently, the effect of N sources on the electrocatalytic performance of the catalysts was explored by comparison with Ni‐SA‐DCD/C and Ni‐SA‐MA/C. The morphology and structure of Ni‐SA‐DCD/C and Ni‐SA‐MA/C were characterized via TEM (**Figure** **4**a–c), XRD (Figure 4d), and Raman (Figure 4e), and the results suggest that atomically dispersed Ni were embedded in the carbon support. Based on the ICP‐MS results, the content of Ni is 0.26 wt% for Ni‐SA‐DCD/C and 0.18 wt% for Ni‐SA‐MA/C, respectively. The CO_2_RR performance of Ni‐SA‐BB/C, Ni‐SA‐DCD/C, and Ni‐SA‐MA/C catalysts was subsequently evaluated.

**Figure 5:**
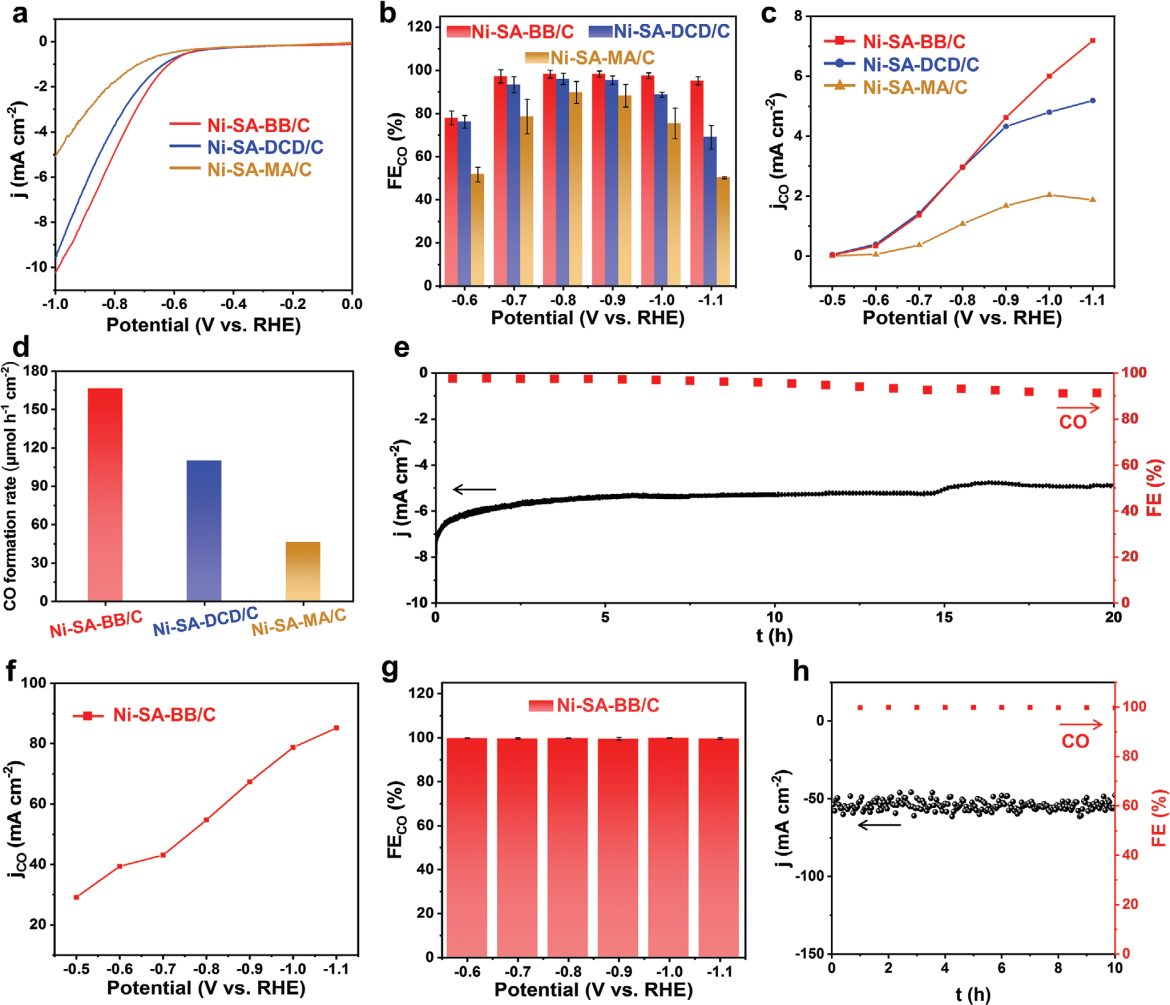
CO_2_ electroreduction performance of Ni‐SA‐BB/C, Ni‐SA‐DCD/C, and Ni‐SA‐MA/C. a) LSV curves in CO_2_‐saturated 0.5 m KHCO_3_ solution. b) Comparison of FE_CO_ at various potentials in H‐cell. c) Comparison of *j*_CO_ at various potentials in H‐cell. d) CO production rate at −1.1 V. e) Long‐term stability test of Ni‐SA‐BB/C for CO_2_ reduction operated at −0.9 V in H‐cell. f) Comparison of *j*_CO_ at various potentials in flow‐cell for Ni‐SA‐BB/C. g) Comparison of FE_CO_ at various potentials in flow‐cell for Ni‐SA‐BB/C. h) Long‐term stability test of Ni‐SA‐BB/C for CO_2_ reduction operated at −0.8 V in flow‐cell.

**Figure** **5**a shows the results from linear sweep voltammetry (LSV) for all samples in a CO_2_‐saturated 0.5 m KHCO_3_ solution. The cathodic current density of Ni‐SA‐BB/C, Ni‐SA‐DCD/C, and Ni‐SA‐MA/C all rise rapidly when the applied potential is lower than −0.6 V. At the same time, both Ni‐SA‐DCD/C and Ni‐SA‐MA/C show much lower current density within the same potential range relative to Ni‐SA‐BB/C, implying the superior catalytic activity of the latter for CO_2_RR. In addition, for the Ni‐SA‐BB/C catalyst in the N_2_‐saturated KHCO_3_ solution, the corresponding current density diminishes markedly due to the relatively poor hydrogen evolution reaction (HER) performance (Figure S7, Supporting Information). To analyze and quantify the generated products, the electrochemical CO_2_RR was performed in the electrolyte solution for 15 min. H_2_ and CO were detected as the major gas products by online gas chromatography, and no liquid products were found by ^1^H NMR spectroscopy (Figure S8, Supporting Information). For Ni‐SA‐DCD/C and Ni‐SA‐MA/C, the maximum faradaic efficiency (FE_CO_) is 94.22% and 86.23% at a moderate potential of −0.8 V, respectively. As the over‐potential increases, the FE_CO_ significantly declines to 72.9% for Ni‐SA‐DCD/C and 34.8% for Ni‐SA‐MA/C at −1.1 V. In contrast, the Ni‐SA‐BB/C electrode shows high selectivity toward CO production in all the investigated potential ranges (Figure 5b). The FE_CO_ for Ni‐SA‐BB/C reaches nearly 100% in a wide potential window from −0.7 to −1.1 V. In addition, the value of FE_CO_ for Ni‐SA‐BB/C (95.6%) is 1.3 and 2.8 times higher than that for Ni‐SA‐DCD/C (72.9%) and Ni‐SA‐MA/C (34.8%) at the high potential of −1.1 V, respectively. As expected, a higher partial current density of CO (*j*_CO_) is obtained for Ni‐SA‐BB/C at various potentials (Figure 5c). Similarly, the largest *j*_CO_ achieved on the Ni‐SA‐BB/C catalyst is 7.2 mA cm^−2^ at −1.1 V, which is much higher than that of Ni‐SA‐DCD/C (5.2 mA cm^−2^) and Ni‐SA‐MA/C (1.9 mA cm^−2^). The high FE_CO_ and large *j*_CO_ directly promote the CO yield. Compared to the low CO production rate for the Ni‐SA‐DCD/C catalyst (109.8 µmol cm^−2^ h^−1^) and Ni‐SA‐MA/C catalyst (46.2 µmol cm^−2^ h^−1^), the Ni‐SA‐BB/C catalyst can reach 166.4 µmol cm^−2^ h^−1^ at a constant potential of −1.1 V (Figure 5d). Taken together, compared with DCD and MA, the selectivity and catalytic activity for CO_2_‐to‐CO are significantly improved when [BMIM][BF_4_] is used as liquid N sources to construct the Ni‐SA catalysts. When [BMIM][BF_4_] was replaced with other ILs ([BMIM][PF_6_] and [EMIM][BF_4_]) during the preparation process, the obtained Ni‐SA catalysts (separately denoted as Ni‐SA‐BP/C and Ni‐SA‐EB/C) still possess similar morphology features as Ni‐SA‐BB/C (Figure S9, Supporting Information) and excellent catalytic activity (Figure S10, Supporting Information). Apart from the good activity, a long‐term electrocatalytic durability is another imperative criterion to appraise advanced catalysts and we here find that after the Ni‐SA‐BB/C catalyst is subjected to a successive 20 h chronoamperometry test at −0.9 V, the current density showed only a slight decay after 15 h, and that the FE_CO_ is well maintained during the continuous test (20 h), verifying the good stability of Ni‐SA‐BB/C (Figure 5e).

Normally, the H‐type cell is employed for CO_2_RR because of the simple structure and convenient assembly, however, CO_2_ is required to be dissolved in the electrolyte before reaction. Due to the low solubility of CO_2_ in aqueous electrolysis, the current density in H‐type cell is suppressed owing to the limited mass transfer process. Thus, a gas‐fed flow cell equipped with gas diffusion electrode was employed to perform the CO_2_RR for Ni‐SA‐BB/C. The current density is greatly increased to 85.21 mA cm^−2^ at −1.1 V (Figure 5f) unfolding the superiority of flow cell, which is 11.8 times that in H‐type cell. And the FE_CO_ is above 99% over a wide potential range from −0.5 to −1.1 V with the maximum value of 99.8% at −0.9 V (Figure 5g). Moreover, current density and FE_CO_ could be sustained at −0.8 V during 10 h electrolysis indicating the good durability of Ni‐SA‐BB/C (Figure 5h).

To highlight the activity of the Ni‐SA‐BB/C catalyst relative to other metal‐SA catalysts, we calculated turnover frequencies (TOFs) per Ni site based on the total Ni content in the Ni‐SA‐BB/C catalyst for CO production at different potentials. Although the TOF value of Ni‐SA‐BB/C catalyst is not the highest among the most active SA catalysts for CO_2_RR, it is even comparable to some state‐of‐the‐art noble metal catalysts, as illustrated in Figure S11 in the Supporting Information. In addition, the performance of Ni‐SA‐BB/C is also comparable to those of many previously reported Ni‐based electrocatalysts—a detailed comparison is summarized in Tables S3 and S4 in the Supporting Information.

Electrochemical surface area (ECSA) and Tafel analysis were performed to get insight into the superior performance of the CO_2_RR. The ECSAs were estimated based on the double‐layer capacitance (*C*_dl_) of the samples to confirm their available surface area during the electrocatalytic process. As shown in Figure S12 in the Supporting Information, the *C*_dl_ of Ni‐SA‐BB/C is about 8.6 mF cm^−2^, which is superior to that of Ni‐SA‐DCD/C (4.9 mF cm^−2^) and Ni‐SA‐MA/C (4.8 mF cm^−2^). The higher ECSA in Ni‐SA‐BB/C stems from the highly exposed active sites (Ni‐N‐C) associated with the liquid environment of the ILs during the synthesis process. These aforementioned features of the ILs can effectively prevent the loss of N sources, leading to more exposed active sites in the Ni‐SA‐BB/C sample. Furthermore, the Tafel analysis was performed to assess the reaction kinetics of the samples. Tafel slopes of Ni‐SA‐BB/C, Ni‐SA‐DCD/C, and Ni‐SA‐MA/C were obtained as 117, 134, and 141 mV dec^−1^, respectively (Figure S13, Supporting Information). The values of the Tafel slope for three catalysts are all close to the theoretical value for the initial one‐electron transfer to CO_2_ (118 mV dec^−1^), revealing that the generation of the adsorbed *COOH intermediate is the rate‐determining step for the overall process,^[^ ^44^ ^]^ something that is further verified via theoretical calculations shown in the next section. It is noted that Ni‐SA‐BB/C displays the minimum Tafel slope among the three samples, implying the fastest initial electron transfer to the CO_2_ molecules to activate them. Hence, the outstanding performance of Ni‐SA‐BB/C can be ascribed to the more activated catalytic sites and the faster electron transfer.

### 2.4 Gram‐Scale Production of Ni‐SA Catalysts

**Figure 6:**
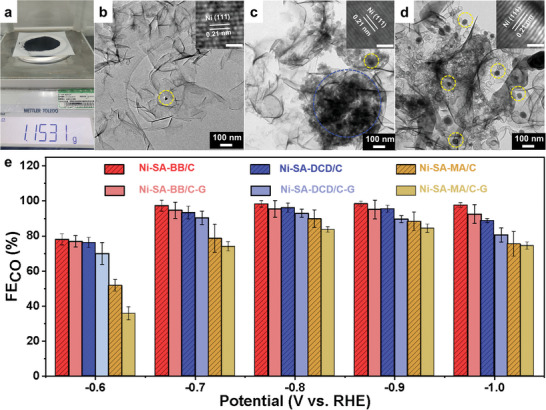
Gram‐scale synthesis of Ni‐SA‐BB/C‐G catalyst. a) Picture of Ni‐SA‐BB/C‐G catalyst with gram‐scale. TEM images of b) Ni‐SA‐BB/C‐G, c) Ni‐SA‐DCD/C‐G, d) Ni‐SA‐MA/C‐G (Inset: the corresponding HR‐TEM image. Scale bars: 1 nm), and e) FE_CO_ of different catalysts (Ni‐SA‐BB/C, Ni‐SA‐DCD/C, Ni‐SA‐MA/C, Ni‐SA‐BB/C‐G, Ni‐SA‐DCD/C‐G, and Ni‐SA‐MA/C‐G) at various potentials.

Although Ni‐SA catalysts display exceptional catalytic performance toward CO_2_RR, currently, the synthesis of Ni‐SA catalysts mostly remains at the milligram levels in the laboratory. Strict conditions (such as multi‐step pyrolysis, template assisting, and acid leaching) are the main factors restricting its large‐scale synthesis.^[^ ^45^ ^]^ How to break through the synthesis problems, realize the gram‐scale even kilogram‐level preparation of Ni‐SA catalysts, and promote the real industrialization of SA catalysts from the laboratory, are still major challenges. In our experiment, the Ni‐SA‐BB/C catalyst, showing highly efficient CO_2_RR, has been obtained via a one‐step pyrolysis and no templates. Based on previous reports, ILs endow a strong polarizing force, which is capable of breaking Ni—Ni bonds and impeding the formation of Ni‐NP.^[^ ^46^ ^]^ Hence, the Ni‐SA‐BB/C catalyst is likely to maintain its excellent catalytic features in the case of amplifying the synthetic scale without acid leaching because only thimbleful Ni‐NP are formed (thus most Ni‐SA are retained) in such a liquid environment. To verify this conjecture, we elevated the synthetic scale of Ni‐SA‐BB/C from milligram level to gram level (1.2 g) without acid leaching, here denoted as Ni‐SA‐BB/C‐G (**Figure** **6**a). In addition, Ni‐SA‐DCD/C and Ni‐SA‐MA/C without acid leaching (milligram levels) were used as contrast samples (denoted as Ni‐SA‐DCD/C‐G and Ni‐SA‐MA/C‐G) and analyzed. TEM images of Ni‐SA‐BB/C‐G (Figure 6b), Ni‐SA‐DCD/C‐G (Figure 6c), and Ni‐SA‐MA/C‐G (Figure 6d) substantiate that Ni‐NP (labeled with dashed yellow circle) can be observed on the surface of the samples and the corresponding HR‐TEM images of Ni‐NP are shown in the inset. The lattice spacing of Ni‐NP is 0.21 nm, which is assignable to the (111) planes of the Ni‐NP. The significant differences in three samples refer to the amount of Ni‐NP and the degree of aggregation. For Ni‐SA‐DCD/C‐G and Ni‐SA‐MA/C‐G, a mass of Ni‐NP is formed during the preparation process. And there is an obvious agglomeration of Ni‐NP (labeled with dashed blue circle) on the surface of Ni‐SA‐DCD/C‐G. Compared to Ni‐SA‐DCD/C‐G and Ni‐SA‐MA/C‐G, only extremely small amounts of Ni‐NP are observed on the Ni‐SA‐BB/C‐G surface, which is further verified via XRD. As shown in Figure S14 in the Supporting Information, XRD patterns of Ni‐SA‐DCD/C‐G and Ni‐SA‐MA/C‐G display a more distinct peak positioning at 44.5° and 51.9° (as labeled by the inverted triangles and assigned to Ni‐NP) in comparison with Ni‐SA‐BB/C‐G. Such results confirm that [BMIM][BF_4_] can effectively prevent the formation of the Ni‐NP and Ni—Ni bonds.

The electrocatalytic CO_2_RR activity of Ni‐SA‐BB/C‐G was investigated using the same experimental conditions. The FE_CO_ is recorded in Figure 6e. As expected, for the as‐prepared Ni‐SA‐DCD/C‐G and Ni‐SA‐MA/C‐G, the FE_CO_ drops at various potentials relative to Ni‐SA‐DCD/C and Ni‐SA‐MA/C. Especially at the low potential of −0.6 V, the FE_CO_ decreases from 78.23% to 65.6% for Ni‐SA‐DCD/C‐G and from 45.88% to 33.59% for Ni‐SA‐MA/C‐G. Although the FE_CO_ at all potentials slightly decreases for Ni‐SA‐BB/C‐G, it still exceeds 90% in a wide potential window from −0.7 to −1.0 V. In addition, the *j*_CO_ for Ni‐SA‐BB/C‐G is superior to that of Ni‐SA‐BB/C at various potentials (Figure S15, Supporting Information). The results show that the gram‐scale prepared Ni‐SA‐BB/C‐G without acid leaching still retains excellent catalytic activity toward CO_2_RR. The rationale behind the above results is that only thimbleful Ni‐NP are formed in such conditions, suggesting that numerous Ni‐N_4_ active sites are retained with the assistance of the ILs.

**Figure 7:**
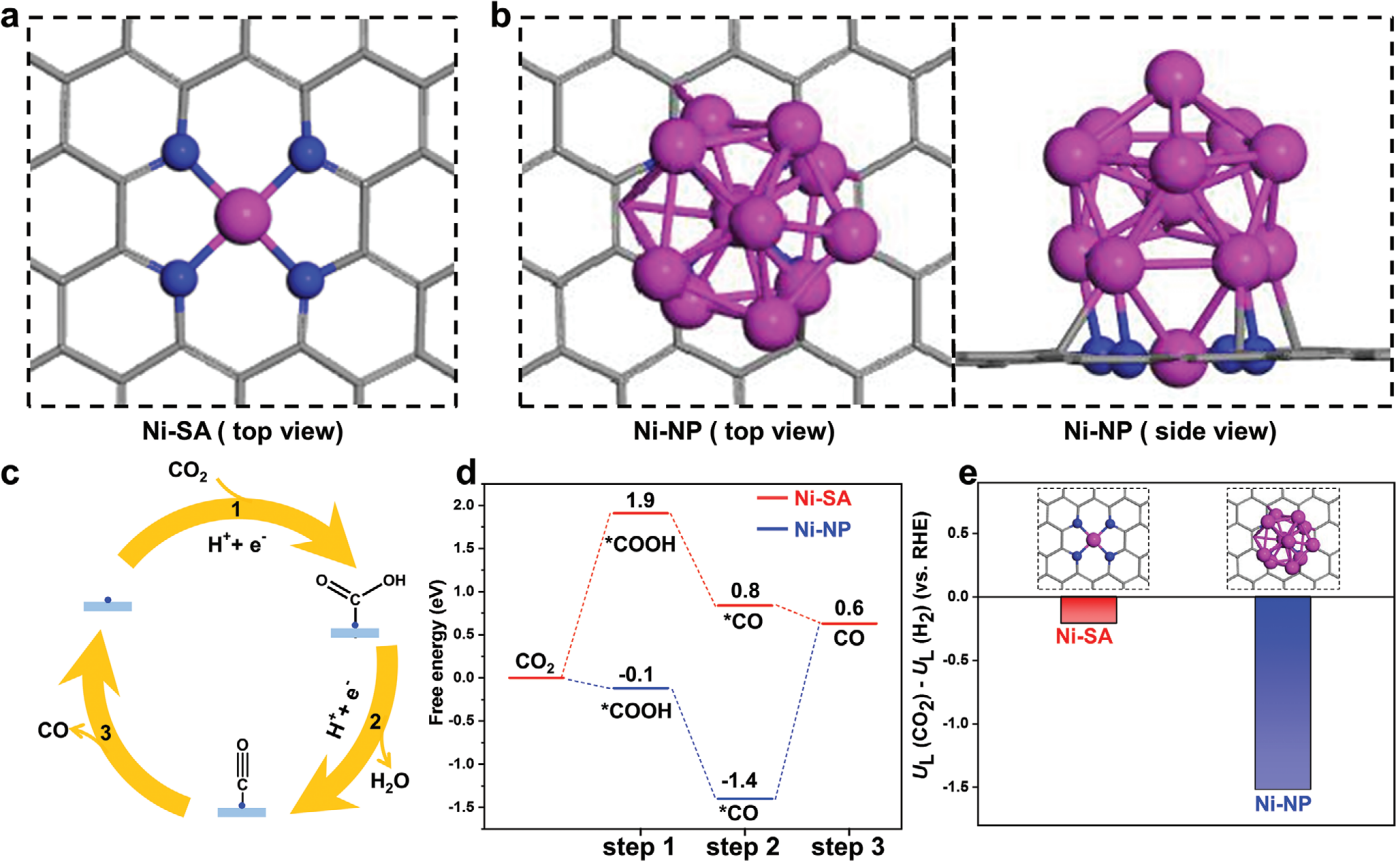
The models of a) Ni‐SA and b) Ni‐NP. c) The typical process of CO_2_ to CO. d) Calculated free energy diagrams for CO_2_RR to CO over different catalyst models. e) The values of *U*_L_(CO_2_)−*U*_L_(H_2_) for Ni‐SA and Ni‐NP.

The underlying role of Ni‐NP on the intrinsic activity of Ni‐SA‐BB/C‐G catalysts was analyzed via density functional theory calculations. The experimental characterization results could establish that the Ni‐SA (**Figure** **7**a) and Ni‐NP (Figure 7b) models are embedded in graphene nanosheets. In addition, a typical CO_2_RR process to CO was described via three elementary steps as follows (Figure 7c). 1) CO_2_ + * +H^+^ +e^−^ → *COOH; 2) *COOH + H^+^ +e^−^ → *CO + H_2_O; and 3) *CO → CO + *.^[^ ^47^ ^]^ Based on the CO_2_RR process, the reaction free energy diagram is displayed in Figure 7d. For the Ni‐SA active site, the formation of *COOH species is an endothermic process—the corresponding energy barrier is predicted to be 1.9 eV in the first step, which can be regarded as the rate‐limiting step. This result is consistent with the Tafel analysis. In contrast, for the Ni‐NP active site, the staging of CO_2_ to *COOH is an exothermic process, and can proceed effortlessly. These results are further proved by calculations of the partial density of states for Ni‐SA and Ni‐NP (Figure S16, Supporting Information). In the second and third steps, showing decreasing free energy profiles, CO can be readily obtained after the *COOH to *CO transition and the desorption of *CO from the active sites for the Ni‐SA active site, whereas the Ni‐NP active site releases *CO to CO with a high energy barrier of 2.0 eV (the rate‐limiting step).

Meanwhile, the energy barrier of the rate‐limiting step on the Ni‐SA active site is lower than that of the Ni‐NP active site, demonstrating that the Ni‐SA active sites have better catalytic performance toward CO_2_RR. It is necessary to point out the different gap between experimental and theoretical results using computational hydrogen electrode models. In the above calculation, the simplest model and ideal environment are considered, The variation of pH values during the electrolysis and applied electrode potential are not considered in the calculation, which are the possible reason resulting in the difference between experimental and theoretical values (see equation in Supporting information). Moreover, the selected model, function, solvent effect, and method all have the effect on the final result. The calculated free energy barrier could not be directly compared with the experimental values, however, it could be utilized to compare the relative activity of various samples.^[^ ^48^, ^49^ ^]^ To further confirm the above result, the CO selectivity was considered as reflected by *U*_L_(CO_2_)−*U*_L_(H_2_) in which *U*_L_(CO_2_) and *U*_L_(H_2_) represent the limiting potentials for the CO_2_RR and HER (Figure 7e), respectively. The *U*_L_(CO_2_)−*U*_L_(H_2_) value for Ni‐SA turns out as more positive than Ni‐NP_,_ indicating the strengthened capability to suppress HER and favor CO_2_RR. The theoretical calculation confirms that the presence of Ni‐NP restrains the catalytic performance of the catalysts toward CO_2_RR. Therefore, compared to Ni‐SA‐DCD/C‐G and Ni‐SA‐MA/C‐G, Ni‐SA‐BB/C‐G contains a minimal amount of Ni‐NP capable of retaining the intrinsic catalytic activity for CO_2_RR.

## 3 Conclusion

A novel, simple, and gram‐scale one‐step pyrolytic method was put forward to construct a nickel single‐atom electrocatalyst with [BMIM][BF_4_] as liquid N sources. Owing to the liquid environment and the space confinement effect of the ionic liquids, the loss of N sources could be much more effectively suppressed compared to earlier studied N sources. Consequently, the as‐synthesized Ni‐SA‐BB/C catalyst exhibits an exceptional FE_CO_ over 95% from −0.7 to −1.1 V, as well as an excellent stability in H‐cell, which well surpasses some earlier prepared Ni‐SA catalysts using conventional N sources. Furthermore, this strategy can realize a gram‐scale preparation of Ni‐SA catalysts even without acid leaching. The gram‐scale prepared Ni‐SA‐BB/C‐G catalyst still retains excellent catalytic activity toward CO_2_RR because only extremely small amounts of Ni‐NP are formed. We believe that this work sheds light and new perspectives on the development of Ni‐SA catalysts with ILs as liquid N sources for applications in the energy storage and conversion areas.

## 4 Experimental Section

### Synthesis of Ni‐SA‐BB/C

In brief, carbon support (75 mg), [BMIM][BF_4_] (1 g), and NiCl_2_⋅6H_2_O (21 mg) were dispersed in 70 mL methanol and sonicated for 10 min. The suspension was stirred at room temperature for 12 h. Subsequently, the mixed solution was heated to 60 °C to thoroughly remove methanol. Then, the black mixture was placed on an alumina boat and pyrolyzed at 1000 °C for 2 h under the Ar atmosphere. After cooling down to room temperature, the black powder was homogenously dispersed in 0.5 m H_2_SO_4_ solution (60 °C) for 12 h. Finally, the sample was collected by centrifugation and washed with deionized water several times until pH ≈7. The collected sample was dried at 60 °C overnight to obtain the final product.

### Large‐Scale Synthesis of Ni‐SA‐BB/C

The step of large‐scale synthesis for Ni‐SA‐BB/C was similar to the above procedure. First, carbon support (1.275 g), [BMIM][BF_4_] (17 g), and NiCl_2_⋅6H_2_O (0.357 g) were dispersed in 1200 mL methanol and sonicated for 10 min. Then, the black mixture was placed on an alumina boat and pyrolyzed at 1000 °C for 2 h under the Ar atmosphere after thoroughly removing methanol. Finally, the sample was collected by centrifugation without acid leaching, which was denoted as Ni‐SA‐BB/C‐G.

## Conflict of Interest

The authors declare no conflict of interest.

## Supporting information

Supporting Information

## Data Availability

The data that support the findings of this study are available in the supplementary material of this article.

## References

[1] S. C. Peter , ACS Energy Lett. 2018, 3, 1557.

[2] W. Ni , Z. Liu , Y. Zhang , C. Ma , H. Deng , S. Zhang , S. Wang , Adv. Mater. 2021, 33, 2003238. 10.1002/adma.202003238 33241569

[3] C. Costentin , M. Robert , J.‐M. Savéant , Chem. Soc. Rev. 2013, 42, 2423. 23232552 10.1039/c2cs35360a

[4] H. B. Savéant , S.‐F. Hung , S. Liu , K. Yuan , S. Miao , L. Zhang , X. Huang , H.‐Y. Wang , W. Cai , R. Chen , J. Gao , X. Yang , W. Chen , Y. Huang , H. M. Chen , C. M. Li , T. Zhang , B. Liu , Nat. Energy 2018, 3, 140.

[5] M. Liu , Y. Pang , B. Zhang , P. De Luna , O. Voznyy , J. Xu , X. Zheng , C. T. Dinh , F. Fan , C. Cao , F. Arquer , T. S. Safaei , A. Mepham , A. Klinkova , E. Kumacheva , T. Filleter , D. Sinton , S. O. Kelley , E. H. Sargent , Nature 2016, 537, 382. 27487220 10.1038/nature19060

[6] M. Li , H. Wang , W. Luo , P. C. Sherrell , J. Chen , J. Yang , Adv. Mater. 2020, 32, 2001848. 10.1002/adma.202001848 32644259

[7] X. Cao , L. Zhao , B. Wulan , D. Tan , Q. Chen , J. Ma , J. Zhang , Angew. Chem., Int. Ed. 2022, 134, e202113918. 10.1002/anie.202113918 34907631

[8] J. Chen , Z. Li , X. Wang , X. Sang , S. Zheng , S. Liu , B. Yang , Q. Zhang , L. Lei , L. Dai , Y. Hou , Angew. Chem., Int. Ed. 2022, 134, e202111683. 10.1002/anie.202111683 34608726

[9] J. Gu , C.‐S. Hsu , L. Bai , H. M. Chen , X. Hu , Science 2019, 364, 1091. 31197014 10.1126/science.aaw7515

[10] S.‐G. Han , D.‐D. Ma , S.‐H. Zhou , K. Zhang , W.‐B. Wei , Y. Du , X.‐T. Wu , Q. Xu , R. Zou , Q.‐L. Zhu , Appl. Catal., B 2021, 283, 119591.

[11] A. S. Varela , N. Ranjbar Sahraie , J. Steinberg , W. Ju , H. S. Oh , P. Strasser , Angew. Chem., Int. Ed. 2015, 54, 10758. 10.1002/anie.201502099 26227677

[12] F. Pan , H. Zhang , K. Liu , D. Cullen , K. More , M. Wang , Z. Feng , G. Wang , G. Wu , Y. Wu , ACS Catal. 2018, 8, 3116.

[13] L. Han , S. Song , M. Liu , S. Yao , Z. Liang , H. Cheng , Z. Ren , W. Liu , R. Lin , G. Qi , X. Liu , Q. Wu , J. Luo , H. L. Xin , J. Am. Chem. Soc. 2020, 142, 12563. 32536159 10.1021/jacs.9b12111

[14] B. Zhang , J. Zhang , J. Shi , D. Tan , L. Liu , F. Zhang , C. Lu , Z. Su , X. Tan , X. Cheng , B. Han , L. Zheng , J. Zhang , Nat. Commun. 2019, 10, 2980. 31278257 10.1038/s41467-019-10854-1 PMC6611886

[15] J. Guo , W. Zhang , L. H. Zhang , D. Chen , J. Zhan , X. Wang , N. R. Shiju , F. Shiju , Adv. Sci. 2021, 8, 2102884. 10.1002/advs.202102884 PMC8655193 34693659

[16] P. Su , K. Iwase , S. Nakanishi , K. Hashimoto , K. Kamiya , Small 2016, 12, 6083. 27634486 10.1002/smll.201602158

[17] C. Zhao , X. Dai , T. Yao , W. Chen , X. Wang , J. Wang , J. Yang , S. Wei , Y. Wu , Y. Li , J. Am. Chem. Soc. 2017, 139, 8078. 28595012 10.1021/jacs.7b02736

[18] X. Li , W. Bi , M. Chen , Y. Sun , H. Ju , W. Yan , J. Zhu , X. Wu , W. Chu , C. Wu , Y. Xie , J. Am. Chem. Soc. 2017, 139, 14889. 28992701 10.1021/jacs.7b09074

[19] K. Gong , F. Du , Z. Xia , M. Durstock , L. Dai , Science 2009, 323, 760. 19197058 10.1126/science.1168049

[20] J. Wu , R. M. Yadav , M. Liu , P. P. Sharma , C. S. Tiwary , L. Ma , X. Zou , X.‐D. Zhou , B. I. Yakobson , J. Lou , P. M. Ajayan , ACS Nano 2015, 9, 5364. 25897553 10.1021/acsnano.5b01079

[21] Q. Zhou , J. Cai , Z. Zhang , R. Gao , B. Chen , G. Wen , L. Zhao , Y. Deng , H. Dou , X. Gong , Y. Zhang , Y. Hu , A. Yu , X. Sui , Z. Wang , Z. Chen , Small Methods 2021, 5, 2100024. 10.1002/smtd.202100024 34927909

[22] Z. Yang , B. Chen , W. Chen , Y. Qu , F. Zhou , C. Zhao , Q. Xu , Q. Zhang , X. Duan , Y. Wu , Nat. Commun. 2019, 10, 3734. 31427572 10.1038/s41467-019-11796-4 PMC6700197

[23] C. Zhao , Y. Wang , Z. Li , W. Chen , Q. Xu , D. He , D. Xi , Q. Zhang , T. Yuan , Y. Qu , J. Yang , F. Zhou , Z. Yang , X. Wang , J. Wang , J. Luo , Y. Li , H. Duan , Y. Li , Joule 2019, 3, 584.

[24] Y. Qu , Z. Li , W. Chen , Y. Lin , T. Yuan , Z. Yang , C. Zhao , J. Wang , C. Zhao , X. Wang , F. Zhou , Z. Zhuang , Y. Wu , Y. Li , Nat. Catal. 2018, 1, 781.

[25] M. Xiao , L. Zhang , B. Luo , M. Lyu , Z. Wang , H. Huang , S. Wang , A. Du , L. Wang , Angew. Chem., Int. Ed. 2020, 132, 7297. 10.1002/anie.202001148 32067299

[26] H. Jing , Z. Zhao , C. Z. , W. Liu , D. Wu , C. Zhu , C. Hao , J. Zhang , Y. Shi , Nano Res. 2021, 14, 4025.

[27] J. P. Paraknowitsch , J. Zhang , D. Su , A. Thomas , M. Antonietti , Adv. Mater. 2010, 22, 87. 20217703 10.1002/adma.200900965

[28] O. Aschenbrenner , S. Supasitmongkol , M. Taylor , P. Styring , Green Chem. 2009, 11, 1217.

[29] P. Kubisa , Prog. Polym. Sci. 2004, 29, 3.

[30] H.‐Y. Jeong , M. Balamurugan , V. S. K. Choutipalli , E.‐s. Jeong , V. Subramanian , U. Sim , K. T. Nam , J. Mater. Chem. A 2019, 7, 10651.

[31] Z. Li , G. Li , L. Jiang , J. Li , G. Sun , C. Xia , F. Li , Angew. Chem., Int. Ed. 2015, 54, 1494. 10.1002/anie.201409579 25504819

[32] H. Wu , J. Song , C. Xie , Y. Hu , B. Han , Green Chem. 2018, 20, 1765.

[33] J. Pei , T. Wang , R. Sui , X. Zhang , D. Zhou , F. Qin , X. Zhao , Q. Liu , W. Yan , J. Dong , L. Zheng , A. Li , J. Mao , W. Zhu , W. Chen , Z. Zhuang , Energy Environ. Sci. 2021, 14, 3019.

[34] Y. Li , S. L. Zhang , W. Cheng , Y. Chen , D. Luan , S. Gao , X. W. Lou , Adv. Mater. 2022, 34, 2105204. 10.1002/adma.202105204 34610187

[35] S. Liu , H. Yang , X. Huang , L. Liu , W. Cai , J. Gao , X. Li , T. Zhang , Y. Huang , B. Liu , Adv. Funct. Mater. 2018, 28, 1800499.

[36] G. Abrasonis , R. Gago , M. Vinnichenko , U. Kreissig , A. Kolitsch , W. Möller , Phys. Rev. B 2006, 73, 125427.

[37] C. Hu , S. Bai , L. Gao , S. Liang , J. Yang , S.‐D. Cheng , S.‐B. Mi , J. Qiu , ACS Catal. 2019, 9, 11579.

[38] H. Fei , J. Dong , C. Wan , Z. Zhao , X. Xu , Z. Lin , Y. Wang , H. Liu , K. Zang , J. Luo , S. Zhao , W. Hu , W. Yan , I. Shakir , Y. Huang , X. Duan , Adv. Mater. 2018, 30, 1802146. 10.1002/adma.201802146 30016001

[39] P. Lu , Y. Yang , J. Yao , M. Wang , S. Dipazir , M. Yuan , J. Zhang , X. Wang , Z. Xie , G. Zhang , Appl. Catal., B 2019, 241, 113.

[40] C. Hering‐Junghans , Angew. Chem., Int. Ed. 2018, 57, 6738. 10.1002/anie.201802675 29718573

[41] X. Liu , L. Xiao , J. Weng , Q. Xu , W. Li , C. Zhao , J. Xu , Y. Zhao , Sci. Adv. 2020, 6, eabb4359. 33177081 10.1126/sciadv.abb4359 PMC7673725

[42] X. Sun , Y. Tuo , C. Ye , C. Chen , Q. Lu , G. Li , P. Jiang , S. Chen , P. Zhu , M. Ma , J. Zhang , Angew. Chem., Int. Ed. 2021, 60, 23614. 10.1002/anie.202110433 34463412

[43] Y. Hou , Y.‐L. Liang , P.‐C. Shi , Y.‐B. Huang , R. Cao , Appl. Catal., B 2020, 271, 118929.

[44] Y.‐H. Fang , Z.‐P. Liu , ACS Catal. 2014, 4, 4364.

[45] X. He , Y. Deng , Y. Zhang , Q. He , D. Xiao , M. Peng , Y. Zhao , H. Zhang , R. Luo , T. Gan , H. Ji , D. Ma , Cell Rep. Phys. Sci. 2020, 1, 100004.

[46] Y. Shi , G. Zhan , H. Li , X. Wang , X. Liu , L. Shi , K. Wei , C. Ling , Z. Li , H. Wang , C. Mao , X. Liu , L. Zhang , Adv. Mater. 2021, 33, 2100143. 10.1002/adma.202100143 34331321

[47] J. Feng , H. Gao , L. Zheng , Z. Chen , S. Zeng , C. Jiang , H. Dong , L. Liu , S. Zhang , X. Zhang , Nat. Commun. 2020, 11, 4341. 32859931 10.1038/s41467-020-18143-y PMC7455739

[48] X. Yang , J. Cheng , X. Xuan , N. Liu , J. Liu , ACS Sustainable Chem. Eng. 2020, 8, 10536.

[49] L. Qiu , S. Shen , C. Ma , C. Lv , X. Guo , H. Jiang , Z. Liu , W. Qiao , L. Ling , J. Wang , Chem. Eng. J. 2022, 440, 135956.

