# Supplementary material for: Liquid Nitrogen Sources Assisting Gram‐Scale Production of Single‐Atom Catalysts for Electrochemical Carbon Dioxide Reduction
Source: Adv Sci (Weinh). 2023 Feb 15;10(11):2205639. doi: 10.1002/advs.202205639 (PMC10104636; doi:10.1002/advs.202205639)
Supplement: Supplementary file 1 — Supporting Information [file ADVS-10-2205639-s001.pdf]

ADVANCED SCIENCE
Open Access

Supporting Information

for *Adv. Sci.*, DOI 10.1002/advs.202205639

Liquid Nitrogen Sources Assisting Gram-Scale Production of Single-Atom Catalysts for Electrochemical Carbon Dioxide Reduction

Beibei An, Jingsheng Zhou, Liangjing Duan, Xiao Liu, Guanyao Yu, Tiegang Ren, Xugeng Guo, Yuanyuan Li, Hans Ågren, Li Wang and Jinglai Zhang**

Supporting Information

Liquid Nitrogen Sources Assisting Gram-scale Production of Single-Atom Catalysts for Electrochemical Carbon Dioxide Reduction

Beibei An,[1,2,3,†] Jingsheng Zhou,[1,2,3,†] Liangjing Duan,[1,2,3,†] Xiao Liu,[1,2,3] Guanyao Yu,[1,2,3] Tiegang Ren,[1,2,3] Xugeng Guo,[1,2,3] Yuanyuan Li,[1,2,3] Hans Ågren,[1,2,3] Li Wang*,[1,2,3] Jinglai Zhang,*,[1,2,3]

[1]*Henan Province Engineering Research Center of Green Anticorrosion Technology for Magnesium Alloys, Henan University, Kaifeng, Henan 475004, P. R. China*
[2]*Henan Engineering Research Center of Corrosion and Protection for Magnesium Alloys, Henan University, Kaifeng, Henan 475004, P. R.China*
[3]*College of Chemistry and Chemical Engineering, Henan University, Kaifeng, Henan 475004, P.R. China*

Author contributions:
[†]Beibei An, Jingsheng Zhou and Liangjing Duan contributed equally to this work.
*Corresponding authors
E-mail addresses: chemwangl@henu.edu.cn (L. Wang) zhangjinglai@henu.edu.cn (J. Zhang).

PartI: Experimental Section

1.1 Materials

Nickel chloride hexahydrate (98%), sodium citrate (98%), 1-butyl-3-methylimidazolium tetrafluoroborate ([BMIM][BF_4], 97%), 1-butyl-3-methylimidazolium hexafluorophosphate ([BMIM][PF_6], 97%), melamine (99%), dicyandiamide (98%), and potassium bicarbonate (99.5%) were purchased from Shanghai Aladdin Biochemical Technology Co. Ltd. Cobalt chloride hexahydrate (AR), tin (II) chloride dihydrate (98%), 1-ethyl-3-methylimidazolium tetrafluoroborate ([EMIM][BF_4], 98%), isopropyl alcohol (≥99.5%) were obtained from Shanghai Macklin Biochemical Co. Ltd. Iron (III) chloride (99%) was purchased from Shanghai Titan Scientific Co. Ltd. Copper (II) chloride dihydrate (99%) and methanol were bought from Tianjin Heowns Biochemical Technology Co. Ltd and Tianjin Fuyu Fine Chemical Co. Ltd, respectively. High purity Argon gas (≥99.999%) and carbon dioxide (≥99.999%) were purchased from Henan Yuanzheng Technology Developement Gas Industry Co. Ltd. All of the chemicals were used directly without further purification. The aqueous solutions were prepared with Millipore water (18.2 MΩ • cm).

1.2 Synthesis of carbon support

The carbon support was synthesized according to the literature with a slight modification [1]. In the typical synthesis, 5 g sodium citrate was heated at 800 °C (10 °C min^{-1}) for 60 min under the Argon (Ar) atmosphere in a tube furnace. Afterward, the black product was dispersed in 0.5 M H_2SO_4 solution and then stirred for 5 h. Finally, the obtained powder was washed with ultrapure water several times and dried at 60 °C in vacuum oven overnight.

1.3 Synthesis of Ni-SA-DCD/C and Ni-SA-MA/C

CN (75 mg), dicyandiamide (DCD) (1 g), and $NiCl_2$•$6H_2O$ (21 mg) were dispersed in 70 mL ethanol and sonicated for 10 min. Then, the suspension was stirred at room temperature for 12 h. Subsequently, the mixed solution was heated and kept at 70 °C to thoroughly remove ethanol. The black powder was placed on an alumina boat and pyrolyzed at 1000 °C for 2 h under an Ar

atmosphere. After cooling down to room temperature, the black powder was homogeneously dispersed in 0.5 M H_2SO_4 solution (60 °C) for 12 h, and washed with deionized water until the pH≈7. The resultant catalyst was donated as Ni-SA-DCD/C. The Ni-SA-MA/C was prepared via a similar process except for DCD was replaced by MA. In addition, the Ni-SA-DCD/C-G and Ni-SA-MA/C-G samples were obtained, which is similar with the procedure to synthesize Ni-SA-BB/C-G.

1.4 Characterizations

The morphology and structure of catalysts were examined by field emission scanning electron microscopy (SEM, FEI Quanta FEG 250) and transmission electron microscopy (TEM, Titan G2 60-300, FEI). The high-angel annular dark-field scanning transmission electron microscope (HAADF-STEM) was performed on FEI Themis Z with a spherical aberration corrector. Powder X-ray diffraction (XRD, Bruker D8 diffractometer) patterns were employed to analyze the crystal structure of the catalysts. Raman spectra were collected using a Raman spectrometer (Renishaw invia) using a 532 nm laser as an excitation source. X-ray photoelectron spectroscopy (XPS) was performed on a Thermo Scientific Escalab-250Xi electron spectrometer using an Al *Kα* X-ray source. The specific surface area and pore size distributions were determined by the Brunauer-Emmett-Teller using an adsorption apparatus (Micromeritics ASAP 2020). The metal content of as-prepared catalysts was determined by inductively coupled plasma mass spectroscopy (ICP-MS) on Agilent 7800. The liquid products were performed by Bruker 300 HZ ^1H nuclear magnetic resonance spectroscopy (NMR) spectroscopy with dimethylsulfoxide (DMSO) as an internal standard. The gas-phase products were analyzed by an on-line gas chromatograph (GC, PANNA A91 Plus) at intervals of 15 min to analyze the concentration of gas products.

1.5 Electrochemical Measurements in H-type cell

An electrochemical workstation (CHI650E) with a gas-tight H-type electrochemical cell separated with a Nafion-117 membrane was used for the electrochemical measurements. A saturated calomel electrode (SCE) and a Pt mesh were used as reference and counter electrodes, respectively. To prepare the working electrode, 5 mg catalysts were dispersed in 480 μl isopropyl

alcohol solution containing 20 μl of 5 wt.% Nafion. Then, the ink was dropped onto a 0.5 × 1 cm^2 carbon paper (HCP020P, from HESEN) to obtain a catalyst area loading of 0.8 mg cm^{-2}. The carbon paper was ultrasonically cleaned in ethanol before being used as the working electrode. The working and the reference electrodes were placed in the cathodic compartment, while the counter electrode was placed in the anodic compartment. Each compartment of the cell was filled with the $KHCO_3$ electrolyte. The electrolyte was purged with N_2 or CO_2 for at least 30 min. During the measurement, CO_2 was continuously bubbled into the cathode compartment at a flow rate of 20 mL min^{-1}. All potentials were converted to the reversible hydrogen electrode (RHE) scale using the equation: $E_{RHE} = E_{SCE} + 0.242$ V+ 0.0592 × pH. The linear sweep voltammetry (LSV) was carried out at a scan rate of 5 mV s^{-1}.

The electrochemical surface area (ECSA) was estimated by measuring the capacitive current associated with electrochemical double-layer capacitance (C_{dl}) from the scan rate dependence of cyclic voltammetry (CV). The measurement was performed among a potential window of -0.4 to -0.5 V vs. SCE where the Faradaic current on the working electrode is negligible.

The gas products were analyzed by a gas chromatograph (GC, A91 Plus PANNA) equipped with a thermal conductivity detector (TCD) and a flame ionization detector (FID). The gas effluent from the cathodic compartment was directly delivered into the gas sampling loop of GC. The Faradaic efficiencies (FEs) of gas products at each applied potential were calculated using the following equation:

$$\mathrm{FE_i} = \frac{2FV_iGtP_0}{RT_0Q_{total}\times 10^6}\times 100\%$$

where V_i (vol %) is the volume concentration of CO in the exhausted gas from the electrochemical cell (GC data) at a given sampling time, G (mL min^{-1}) is the gas flow rate at room temperature and ambient pressure, t (min) is electrolysis time, P_0 is pressure (1.01 × 10^5 Pa), R is the gas constant (8.314 J mol^{-1} K^{-1}), T_0 is the temperature (298.15 K), Q_{total} (C) is integrated charge passed during electrolysis (Chronoamperometry data), F is the Faradaic constant (96485 C mol^{-1}).

The partial current density of CO (j_{CO}) production was calculated using the equation below:

$$j_{CO} = FE_{CO} \times j$$

The turnover frequency (TOF) value of the electrocatalyst was calculated according to the following formula:

$$TOF = \frac{I_{product}/NF}{m_{cat} \times \omega / M_{metal}} \times 3600$$

$I_{product}$ is the partial current for certain product (CO), N is the number of electrons transferred for product formation, in which it is 2 for CO, F is Faradaic constant (96485 C mol^{-1}), m_{cat} is catalyst mass in the electrode, ω is metal loading in the catalyst, M_{metal} is atomic mass of metal.

1.6 Electrochemical measurements in flow cell

A three-chamber electrochemical cell (including CO_2 chamber, catholyte chamber, and anolyte chamber) was used for the flow cell electrochemical measurements. An Ag/AgCl electrode and an iridium oxide supported on titanium mesh (IrO_2/Ti mesh) were applied as the reference electrode and counter electrode, respectively. To prepare the working electrode, 5 mg catalysts were dispersed in 480 μl isopropylalcohol solution containing 20 μl of 5 wt.% Nafion. Then, the ink was dropped onto a gas diffusion layer (0.5 × 2 cm^2) to obtain a catalyst area loading of 1 mg cm^{-2}. Each chamber had an inlet and an outlet for electrolyte or CO_2 gas, and the reference electrode was fixed in the catholyte chamber. The catholyte and anolyte chamber were separated by an anion exchange membrane (Fumasep, FAB-PK-130). 1 M KOH solution was used as both catholyte and anolyte. CO_2 was continuously delivered into the back chamber of catholyte chamber at a flow rate of 20 mL min^{-1} by a mass flow controller.

1.7 X-ray absorption fine structure (*XAFS) measurements*

The XAFS spectra were collected at the 1W1B station in BSRF (Beijing Synchrotron Radiation Facility, China) operated at 2.5 GeV with a maximum current of 250 mA. XAFS measurements at the Ni K edge were performed in the fluorescence excitation mode using a Lytle detector.

Part II: Computation Section

Computational Methods

All calculations were performed using the Vienna Ab initio Simulation Package (VASP) at the spin polarization density functional theory (DFT) level, with the GGA-PBE functional to describe the electron exchange correlation functional. In order to take into account the van der Waals (vdW) effects, the Grimme method (DFT-D3) was included in the calculations. The Hubbard U (DFT + U) correction for the strong-correlation d-electrons of transition metals was considered. The $U_{eff, Ni}$ of Ni 3d orbital was set to be 3.4 eV. The plane wave energy cutoff was set to be 500 eV. A set of (1 × 1 × 1) k-points was carried out for geometric optimization while higher quality of 12 × 12 × 1 was used for partial density of states (PDOS) calculations, and electronic energy and the force convergence criteria were set to be 10^{-5} eV and 0.05 eV $Å^{-1}$, respectively. For the systems, the change of Gibbs free energy (ΔG) during CO_2 transformation was calculated as $\Delta G = \Delta E + \Delta E_{ZPE} + \int C_p dT - T\Delta S + \Delta G_{pH} + \Delta G_U$. Here, ΔE is the electron energy difference calculated by DFT, ΔE_{ZPE}, $\int C_p dT$, and TΔS are the change in zero point energy, enthalpy change, and entropy change, respectively. ΔG_{pH} can be expressed $\Delta G_{pH} = 2.303 \bullet k_B \bullet T \bullet pH$, and the pH value is set to be 0 in this work. ΔG_U can be obtained $\Delta G_U = -n \bullet e \bullet U$, where n is the number of electrons transferred, e is the electron charge, and U is the applied electrode potential.

Part III: Supplementary Results

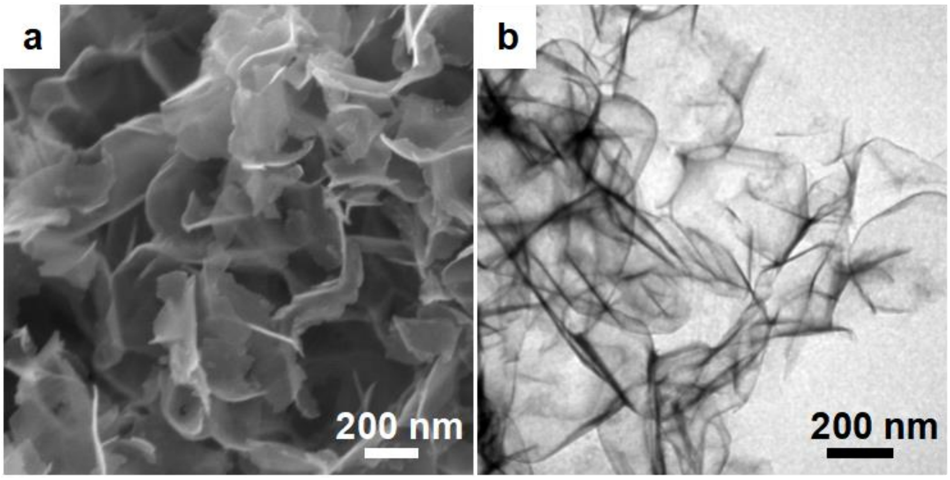

Figure S1. (a) SEM image and (b) TEM image of the carbon support.

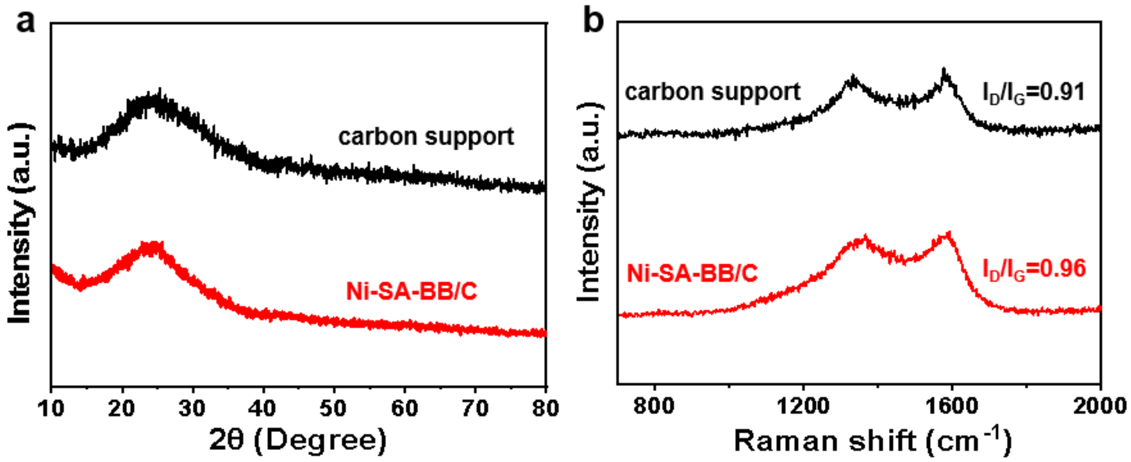

Figure S2. (a) XRD patterns of the carbon support and Ni-SA-BB/C. (b) Raman patterns of the carbon support and Ni-SA-BB/C.

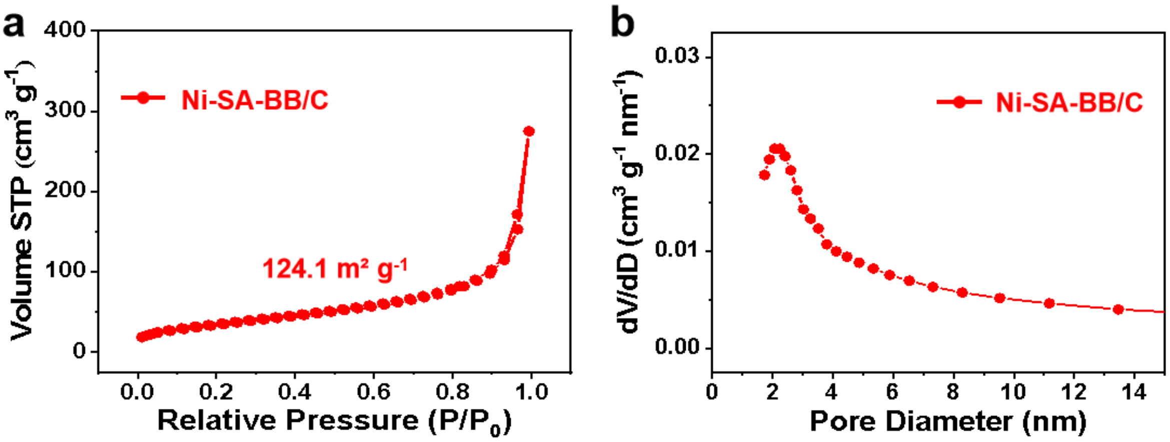

Figure S3. (a) N_2 adsorption-desorption isotherms and (b) pore size distribution of Ni-SA-BB/C.

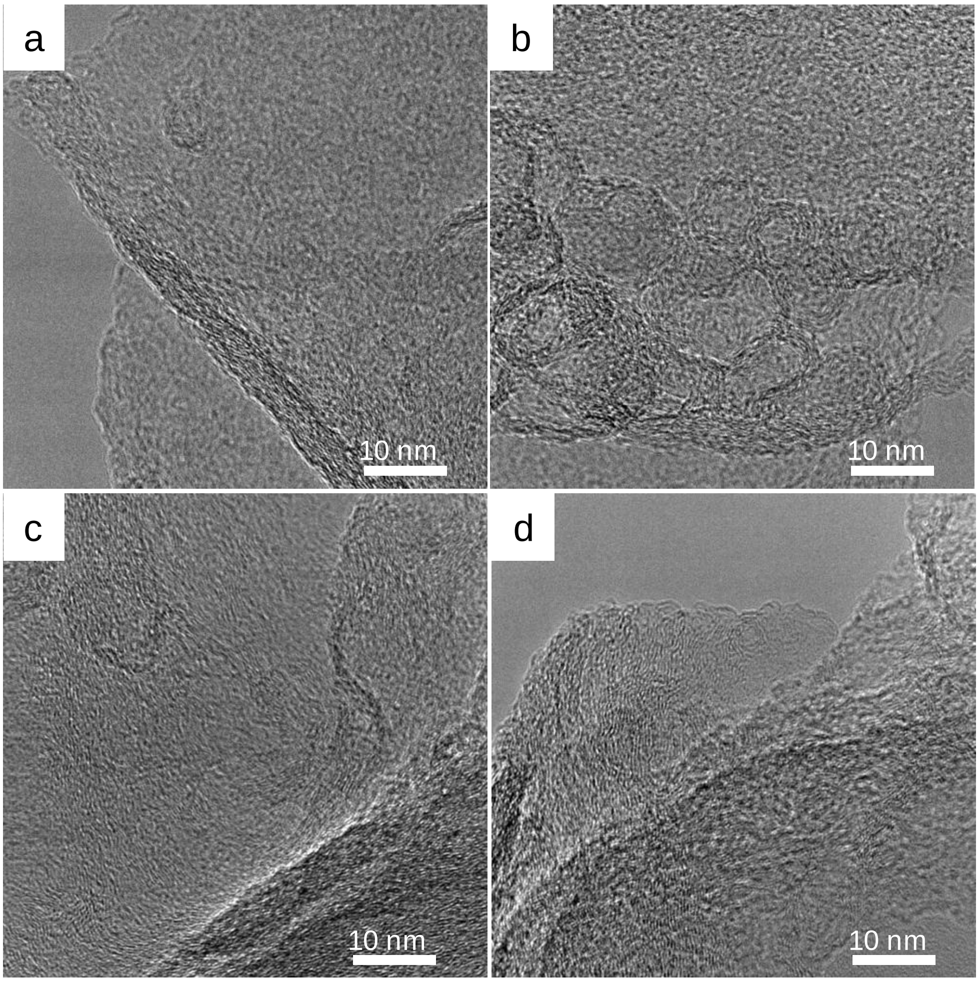

Figure S4. HR-TEM images of (a) Co-SA-BB/C, (b) Cu-SA-BB/C, (c) Fe-SA-BB/C, and (d) Sn-SA-BB/C.

Figure S4 are the HR-TEM images of as-prepared Co-SA-BB/C, Cu-SA-BB/C, Fe-SA-BB/C, and Sn-SA-BB/C, respectively. There are no corresponding metal nanoparticles, metal oxides, and other impurities on the sample surface. However, based on ICP-MS results, the corresponding metal content in Co-SA-BB/C, Cu-SA-BB/C, Fe-SA-BB/C, and Sn-SA-BB/C samples are 0.21, 0.20, 0.13, and 0.37, respectively, as shown in Table S2. Such results imply that corresponding mental dominantly presented the atomic dispersion were anchored in the carbon support for these samples.

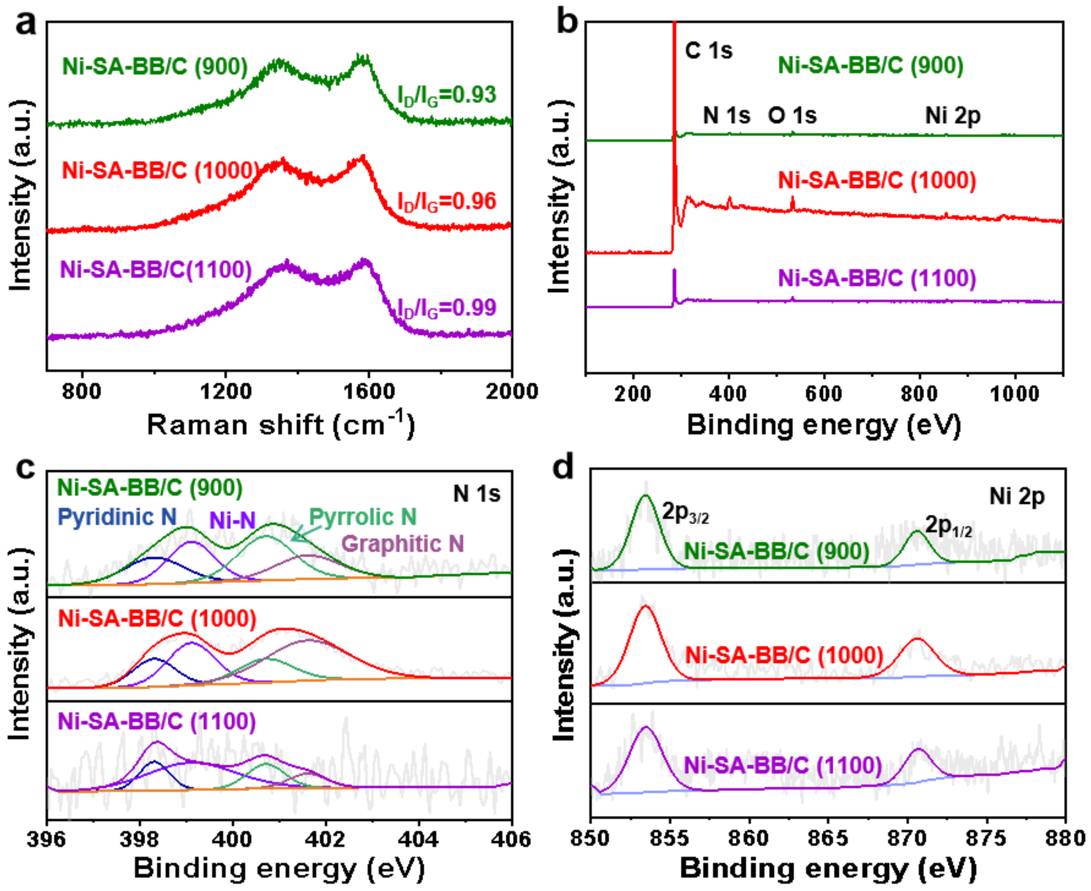

Figure S5. (a) Raman patterns, (b) Full XPS spectra, (c) N 1s HR-XPS spectra, and (d) Ni 2p HR-XPS spectra of Ni-SA-BB/C (900), Ni-SA-BB/C (1000) and Ni-SA-BB/C (1100), respectively.

The effect of pyrolysis temperature on the property and electrochemical CO_2 reduction performance of samples was investigated with the temperature varying from 900 to 1100 °C. The synthesized samples were denoted as Ni-SA-BB/C (900), Ni-SA-BB/C (1000), and Ni-SA-BB/C (1100), respectively. Based on XPS results, the N content in three samples continuously decreases (5.4 at.%→4.4 at.%→2.1 at.%) with increasing pyrolysis temperature, on the contrary, there is a positive correlation between the Ni content (0.71 at.%→0.77 at.%→0.90 at.%) and the pyrolysis temperature. In addition, the I_D/I_G ratio obtained from Raman spectra increases in a small range of 0.93-0.99, suggesting more structural defects of carbon supports. Theoretically, higher pyrolysis temperature leads to loss of N due to C-N cleavages, producing more defects in the carbon supports and promoting the formation of protected Ni-NP (some Ni-NP cannot be removed via acid leaching due to carbon shell protection). Although the existence of defects is beneficial for the CO_2RR, the carbon densification and sintering are caused by extremely high pyrolysis temperature, which leads to the surface area dramatically dropping, excessive loss of nitrogen sources, and aggregation of Ni-NP [2, 3]. Hence, high pyrolysis temperature will have an adverse effect on the performance of CO_2RR, which is in accordance with the results of catalytic experiment, as shown in Figure S6.

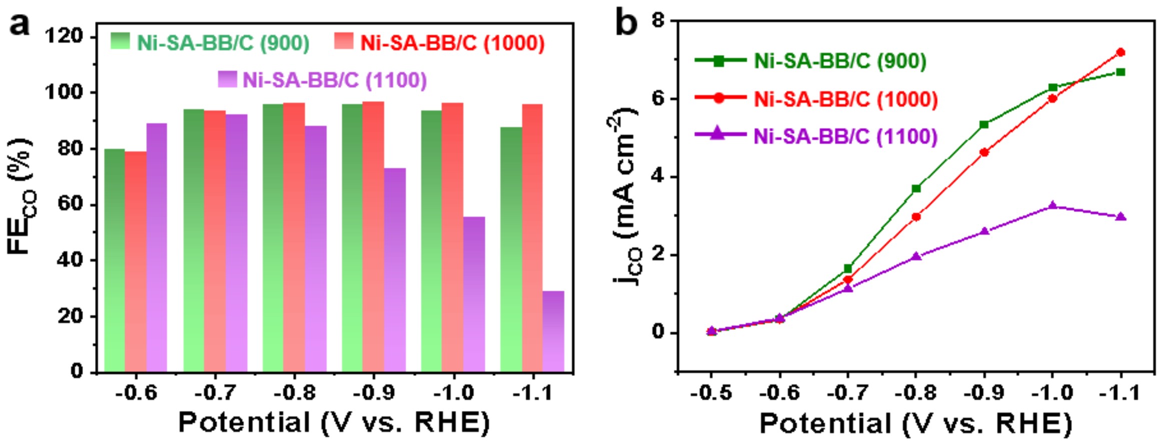

Figure S6. Catalytic performance of Ni-SA-BB/C (900), Ni-SA-BB/C (1000), and Ni-SA-BB/C (1100). (a) FE_{CO} at different applied potentials. (b) j_{CO} at different applied potentials.

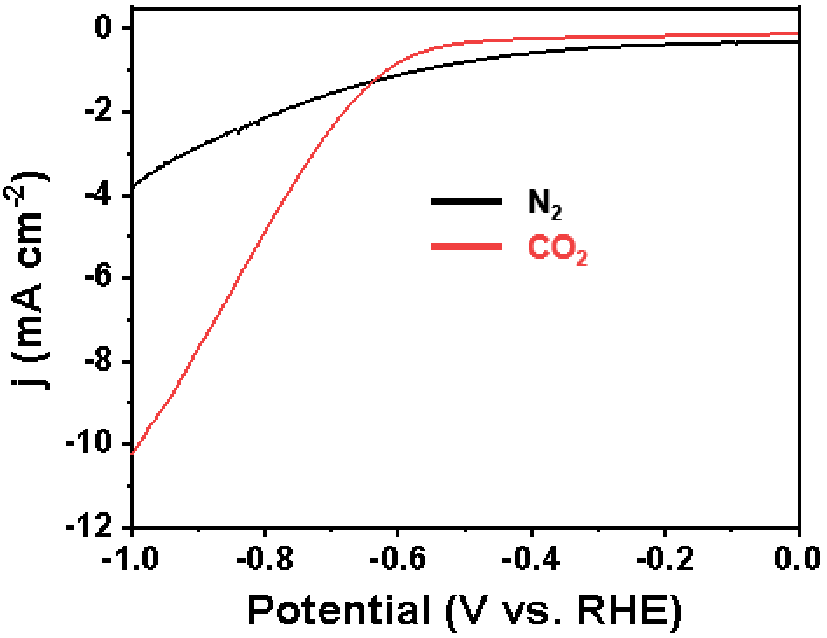

Figure S7. LSV curves of Ni-SA-BB/C in N_2-saturated and CO_2-saturated 0.5 M $KHCO_3$, respectively.

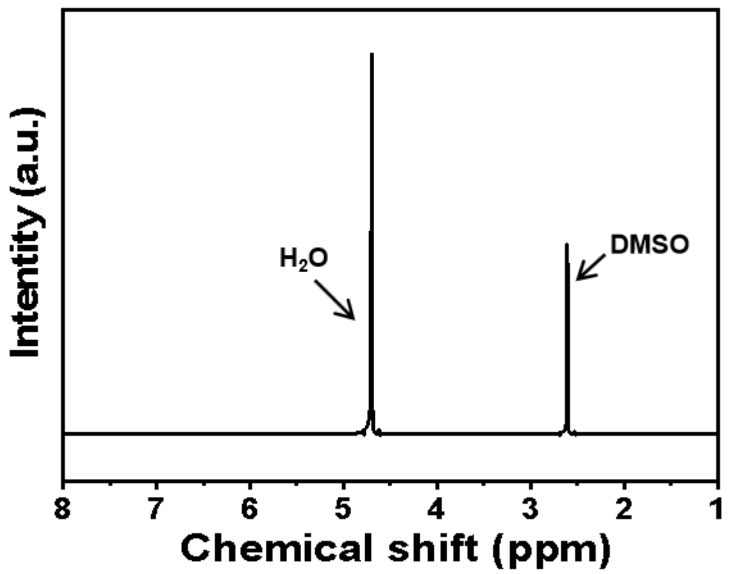

Figure S8. ^{1}H NMR spectrum of electrolysis liquid product for Ni-SA-BB/C.

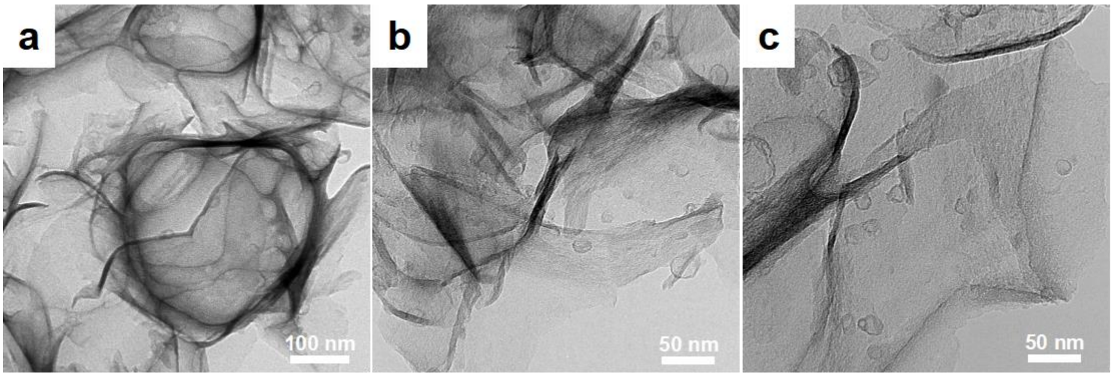

Figure S9. TEM images of (a) Ni-SA-BB/C, (b) Ni-SA-BP/C, and (c) Ni-SA-EB/C.

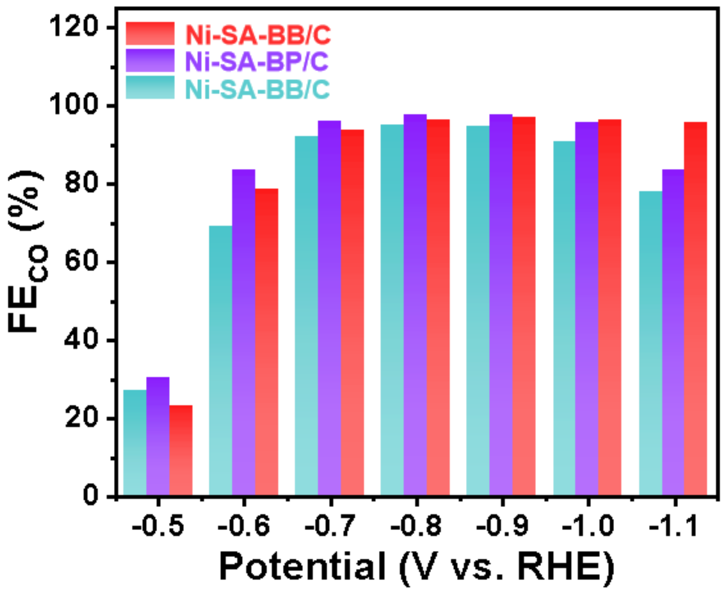

Figure S10. FE_{CO} of Ni-SA-BB/C, Ni-SA-BP/C, and Ni-SA-EB/C.

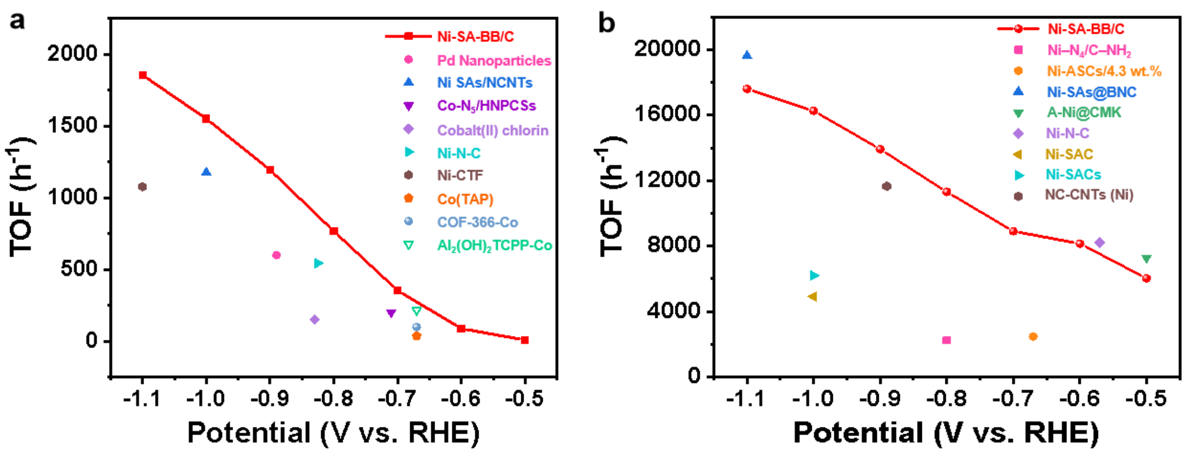

Figure S11. TOF of Ni-SA-BB/C compared with those CO_2-to-CO reduction catalysts in H-cell (a) and flow cell (b) [4-20].

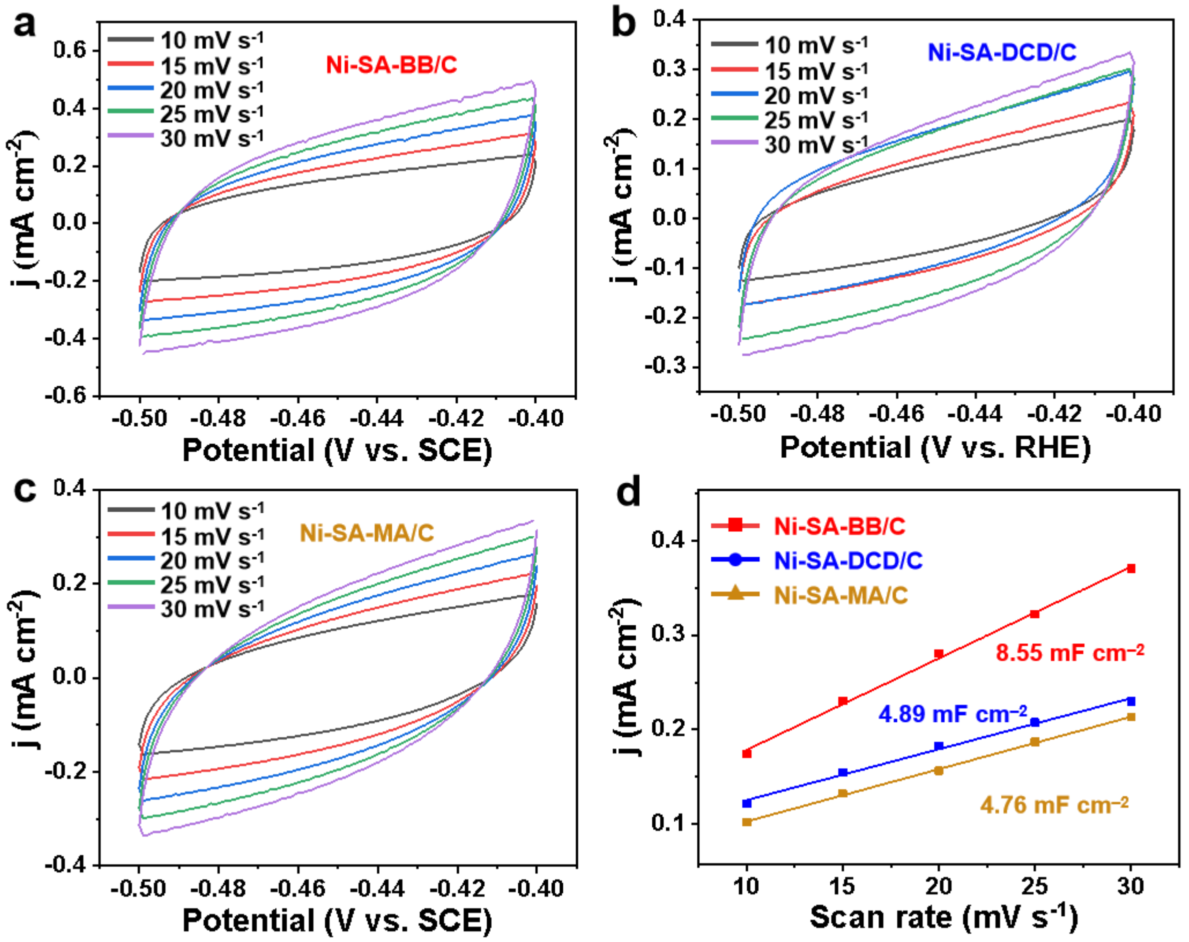

Figure S12. Cyclic voltammograms (CV) curves under different scan rates for (a) Ni-SA-BB/C, (b) Ni-SA-DCD/C, and (c) Ni-SA-MA/C. (d) Charging current density differences j plotted against scan rates for the different catalysts.

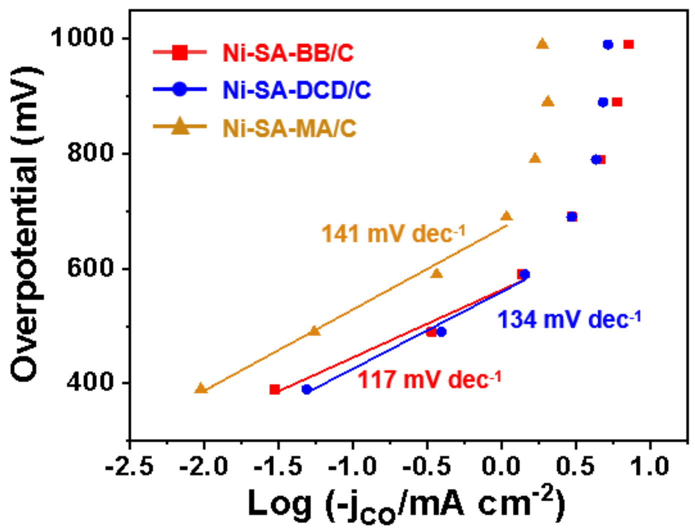

Figure S13. Tafel plots for the CO partial current density of Ni-SA-BB/C, Ni-SA-DCD/C, and Ni-SA-MA/C.

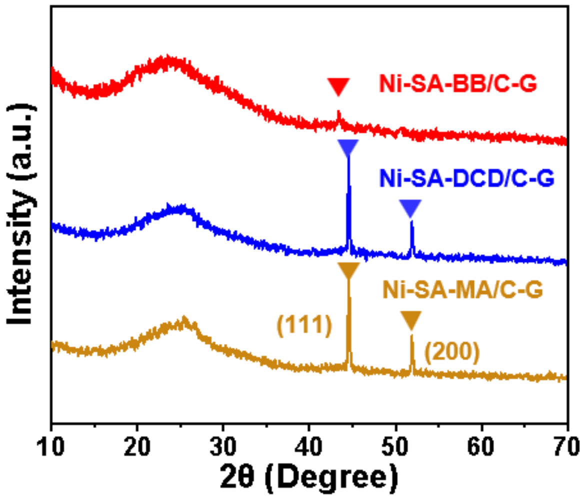

Figure S14. XRD patterns of Ni-SA-BB/C-G, Ni-SA-DCD/C-G, and Ni-SA-MA/C-G.

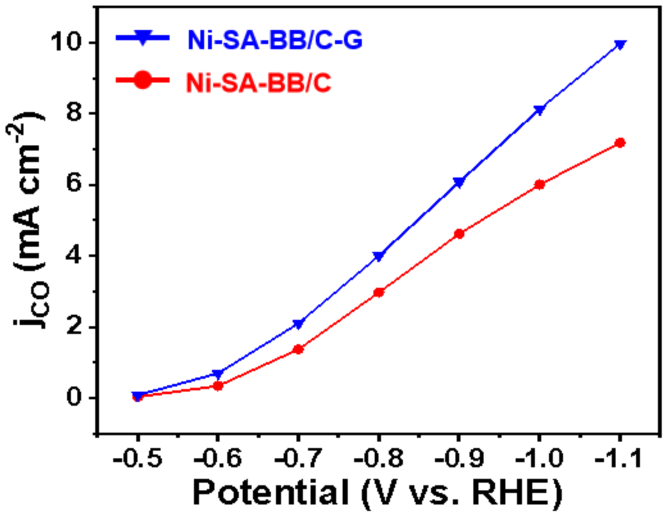

Figure S15. j_{CO} of Ni-SA-BB/C-G and Ni-SA-BB/C at different applied potentials.

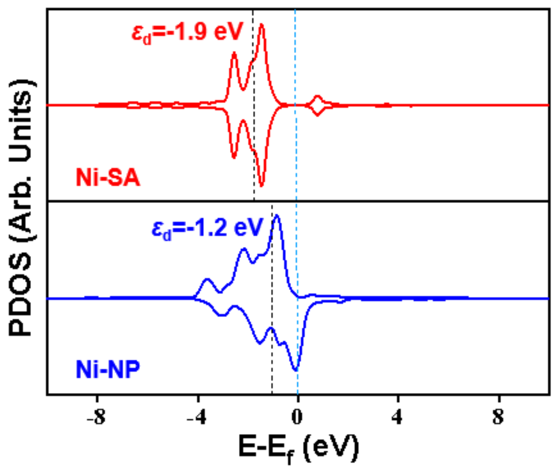

Figure S16. PDOS of Ni 3d in Ni-SA and Ni-NP.

Table S1. Structural parameters of Ni-SA-BB/C extracted from the EXAFS fitting. (S_0^2=0.80)

Sample	Shell	CN	R (Å)	σ^2 (Å^2)	ΔE_0 (eV)	R factor
Ni-SA-BB/C	Ni-N	3.9±0.5	1.90±0.01	0.0077±0.0023	-5.6±1.9	0.017

S_0^2 is the amplitude reduction factor S_0^2=0.8; CN is the coordination number; R is interatomic distance (the bond length between central atoms and surrounding coordination atoms); σ^2 is Debye-Waller factor (a measure of thermal and static disorder in absorber-scatterer distances); ΔE_0 is edge-energy shift (the difference between the zero kinetic energy value of the sample and that of the theoretical model). R factor is used to value the goodness of the fitting.

Table S2. The contents of the metal elements in corresponding samples.

Sample	Metal content (wt. %) (ICP-MS)
Co-SA	0.21
Cu-SA	0.20
Fe-SA	0.13
Sn-SA	0.37

Table S3. Comparison of the Ni single atom catalyst with other Ni catalysts for CO_2 electroreduction in the H-type cell.

Catalysts	N sources	Electrolyte	FE_{CO} (%)	j (mA cm^{-2})	Ref.
Ni SAs N/C	ZIF-8	0.5 M $KHCO_3$	71.9	-10.48	[21]
Ni-N-C	Urea	0.1 M $KHCO_3$	96	-8.2	[22]
Ni-NG	NH_3	0.5 M $KHCO_3$	95	-11	[23]
Ni-N-Gr	Pentaethylenehexamine	0.5 M $KHCO_3$	95	-1.5	[24]
SE-Ni SAs@PNC	ZIF-8	0.5 M $KHCO_3$	96	-7.8	[25]
Ni-N-MEGO	Urea&NH_3	0.5 M $KHCO_3$	92	-20	[26]
C-Zn_1Ni_4	ZIF-8	0.5 M $KHCO_3$	92	-65.7	[27]
Ni-SA-BB/C	[BMIM][BF_4]	0.5 M $KHCO_3$	96.8	-7.2	This work

Table S4. Comparison of the Ni single atom catalyst with other Ni catalysts for CO_2 electroreduction in the flow cell.

Catalysts	N sources	Electrolyte	FE_{CO} (%)	j (mA cm^{-2})	Ref.
Ni-N_4/C-NH_2	ZIF-8	1 M KOH	89.3	-480	[13]
Ni-N_1-C_3	MET-6	1 M KOH	99	-207	[28]
Ni@C_3N_4-CN	C_3N_4-CN NS	1 M $KHCO_3$	96.8	-300	[29]
Ni-NAC	ethylenediamine	0.5 M $KHCO_3$	98	-151	[30]
Ni SAs-NCW	Urea	1 M KOH	95.4	-41.6	[31]
Ni-ASCs	Urea	1 M KOH	95.1	-507.2	[14]
Ni-SAs@BNC	NH_3	0.5 M $KHCO_3$	97	-214	[15]
Ni-SA-BB/C	[BMIM][BF_4]	1 M KOH	99.8	-85.2	This work

References

[1] X. Yang, J. Cheng, X. Xuan, N. Liu, J. Liu, ACS Sustain. Chem. Eng. **2020**, 8, 10536.

[2] P.P. Sharma, J. Wu, R.M. Yadav, M. Liu, C.J. Wright, C.S.Tiwary, B.I. Yakobson, J. Lou, P. M. Ajayan, X.D. Zhou, Angew. Chem. Int. Ed. **2015**, 127, 13905.

[3] H. Li, N. Xiao, M. Hao, X. Song, Y. Wang, Y. Ji, C. Liu, C. Li, Z. Guo, F. Zhang, J. Qiu, Chem. Eng. J. **2018**, 351, 613.

[4] D. Gao, H. Zhou, J. Wang, S. Miao, F. Yang, G. Wang, J. Wang, X. Bao, J. Am. Chem. Soc. **2015**, 137, 4288.

[5] P. Lu, Y. Yang, J. Yao, M. Wang, S. Dipazir, M. Yuan, J. Zhang, X. Wang, Z. Xie, G. Zhang, Appl. Catal. B Environ. **2019**, 241, 113.

[6] Y. Pan, R. Lin, Y. Chen, S. Liu, W. Zhu, X. Cao, W. Chen, K. Wu, W.-C. Cheong, Y. Wang, et al. J. Am. Chem. Soc. **2018**, 140, 4218.

[7] S. Aoi, K. Mase, K. Ohkubo, S. Fukuzumi, Chem. Commun. **2015**, 51, 10226.

[8] W. Ju, A. Bagger, G.-P. Hao, A.S. Varela, I. Sinev, V. Bon, B. Roldan Cuenya, S. Kaskel, J. Rossmeisl, P. Strasser, Nat. Commun. **2017**, 8, 1.

[9] P. Su, K. Iwase, T. Harada, K. Kamiya, S. Nakanishi, Chem. Sci. **2018**, 9, 3941.

[10] N. Han, Y. Wang, L. Ma, J. Wen, J. Li, H. Zheng, K. Nie, X. Wang, F. Zhao, Y. Li, et al. Chem **2017**, 3, 652.

[11] N. Kornienko, Y. Zhao, C.S. Kley, C. Zhu, D. Kim, S. Lin, C.J. Chang, O.M. Yaghi, P. Yang, J. Am. Chem. Soc. **2015**, 137, 14129.

[12] S. Lin, C.S. Diercks, Y.-B. Zhang, N. Kornienko, E.M. Nichols, Y. Zhao, A.R. Paris, D. Kim, P. Yang, O.M. Yaghi, Science **2015**, 349, 1208.

[13] Z. Chen, X. Zhang, W. Liu, M. Jiao, K. Mou, X. Zhang, L. Liu, Energy Environ. Sci. **2021**, 14, 2349-2356.

[14] S. Wang, Z. Qian, Q. Huang, Y. Tan, F. Lv, L. Zeng, C. Shang, K. Wang, G. Wang, Y. Mao, et al. Adv. Energy Mater. **2022**, 2201278.

[15] X. Gu, Y. Jiao, B. Wei, T. Xu, P. Zhai, Y. Wei, J. Zuo, W. Liu, Q. Chen, Z. Yang, et al. Mater. Today **2022**, 54, 63-71.

[16] B. Chen, B. Li, Z. Tian, W. Liu, W. Liu, W. Sun, K. Wang, L. Chen, J. Jiang, Adv. Energy Mater. **2021**, 11, 2102152.

[17] Y. Li, N. M. Adli, W. Shan, M. Wang, M. J. Zachman, S. Hwang, H. Tabassum, S. Karakalos, Z. Feng, G. Wang, et al. Energy Environ. Sci. **2022**, 15, 2108‑2119.

[18] C. Wang, X. Hu, X. Hu, X. Liu, Q. Guan, R. Hao, Y. Liu, W. Li, Appl. Catal. B Environ. **2021**, 296, 120331.

[19] Q. Fan, P. Gao, S. Ren, Y. Qu, C. Kong, J. Yang, Y. Wu, Nano Res. **2022**, 1-8.

[20] Q. Fan, P. Hou, C. Choi, T. S. Wu, S. Hong, F. Li, Y. L. Soo, P. Kang, Y. Jung, Z. Sun, Adv. Energy Mater. **2020**, 10, 1903068.

[21] C. Zhao, X. Dai, T. Yao, W. Chen, X. Wang, J. Wang, J. Yang, S. Wei, Y. Wu, Y. Li, J. Am. Chem. Soc. **2017**, 139, 8078.

[22] F. Pan, W. Deng, C. Justiniano, Y. Li, Appl. Catal. B Environ. **2018**, 226, 463.

[23] K. Jiang, S. Siahrostami, T. Zheng, Y. Hu, S. Hwang, E. Stavitski, Y. Peng, J. Dynes, M. Gangisetty, D. Su, et al. Energy Environ. Sci. **2018**, 11, 893.

[24] P. Su, K. Iwase, S. Nakanishi, K. Hashimoto, K. Kamiya, Small **2016**, 12, 6083.

[25] J. Yang, Z. Qiu, C. Zhao, W. Wei, W. Chen, Z. Li, Y. Qu, J. Dong, J. Luo, Z. Li, et al. Angew. Chem. Int. Ed. **2018**, 57, 14095.

[26] Y. Cheng, S. Zhao, H. Li, S. He, J.-P. Veder, B. Johannessen, J. Xiao, S. Lu, J. Pan, M.F. Chisholm, et al. Appl. Catal. B. Environ. **2019**, 243, 294.

[27] C. Yan, H. Li, Y. Ye, H. Wu, F. Cai, R. Si, J. Xiao, S. Miao, S. Xie, F. Yang, et al. Energy Environ. Sci. **2018**, 11, 1204.

[28] F. Yang, H. Yu, Y. Su, J. Chen, S. Chen, Z. Zeng, S. Deng, J. Wang, Nano Res. **2022**, 1-9.

[29] Q. Wang, K. Liu, K. Hu, C. Cai, H. Li, H. Li, M. Herran, Y.R. Lu, T.S. Chan, C. Ma, et al. Nat. Commun. **2022**, 13, 1-10.

[30] Z. Luo, Z. Yin, J. Yu, Y. Yan, B. Hu, R. Nie, A. Kolln, X. Wu, R. Behera, M. Chen, et al. Small **2022**, 2107799.

[31] H. Chang, H. Pan, F. Wang, Z. Zhang, Y. Kang, S. Min, Nanoscale **2022** 14, 10003-10008.
